# Alleviating effect of *Lactobacillus rhamnosus* SDSP202418 on exercise-induced fatigue in mice

**DOI:** 10.3389/fmicb.2024.1420872

**Published:** 2024-09-26

**Authors:** Yang Yang, Yuanji Zhao, Huan Lei

**Affiliations:** ^1^College of Physical Education, Chengdu Sport University, Chengdu, China; ^2^School of Physical Education, Wuhan Sports University, Wuhan, China

**Keywords:** probiotics, *Lactobacillus rhamnosus* SDSP202418, antifatigue, oxidative stress, muscle fiber, intestinal flora

## Abstract

In this study, the effects of *Lactobacillus rhamnosus* SDSP202418 isolated from shrimp paste on the exercise performance of fatigued mice were analyzed, and the potential action mechanism was revealed. *L. rhamnosus* SDSP202418 significantly improved the exhaustion time of the mice and regulated the biochemical indices (lactate dehydrogenase, nitrogen, and uric acid) of the fatigued mice to resist fatigue. *L. rhamnosus* SDSP202418 also upregulated the mRNA expression of slow muscle fibers and downregulated the mRNA expression of fast muscle fibers in the exercise mice by activating the AMPK/PGC-1α pathway in the fatigued mice. It also increased the contents of antioxidant enzymes (superoxide dismutase (SOD), catalase (CAT), and glutathione (GSH)) in the liver and muscle. These enzymes removed and repaired oxidative free radicals to achieve antifatigue. In addition, *L. rhamnosus* SDSP202418 can change the gut microbial structure and modulate the abundance and balance of fatigue-related gut microbiota, which in turn exerts antifatigue effects. *L. rhamnosus* SDSP202418 is a functional food component that relieves fatigue after exercise.

## 1 Introduction

Exercise fatigue is a physiological phenomenon that occurs after the body has completed a certain amount of mental and physical activity. The body’s physiological processes are unable to maintain their functions at a specific level and intensity (Liu et al., 2022). Excessive fatigue is detrimental to the improvement of athletic performance and may have harmful effects on physical and mental health (Zhang et al., 2019). At present, the physiological mechanism underlying the production of exercise-induced fatigue is primarily summarized as energy and material consumption theory, metabolite accumulation and blockage theory, and protective inhibition theory (Spector et al., 1995; Verkerke et al., 2005; Weisleder and Ma, 2008). High-intensity exercise increases the consumption of energy sources, such as serum and muscle, and the accumulation of metabolites, such as lactic acid (LA), ammonia, and blood urea nitrogen (BUN) (Nikolaidis et al., 2018). Additionally, free radical accumulation caused by exercise-induced fatigue leads to muscle damage and muscle fatigue by triggering lipid peroxidation and disrupting the antioxidant defense system (Gonzalez et al., 2016). The gut is an important organ for maintaining healthy homeostasis, and excessive fatigue can also lead to gut microbial disruption, exacerbating fatigue-induced physical injuries. The relationship between the gut microbiota and exercise fatigue has been extensively studied. An imbalance of intestinal flora and an increased number of pathogenic bacteria were observed in exhaustively exercised mice (Li et al., 2022). The disorders of gut microbes can also cause oxidative stress and exacerbate fatigue. Zhu et al. (2022) found that the Maca compound could increase the number of beneficial bacteria species (*Lactobacillus* and *Akkermansia*) and decrease the number of harmful bacteria species (i.e., *Candidatus_Planktophila* and *Candidatus_Arthromitus*) to modulate gut flora at the genus level. Moreover, a high correlation between fatigue-related biomarkers and fecal microbiota was found. Therefore, fatigue can be ameliorated by maintaining a balanced gut flora.

Delaying or eliminating exercise fatigue is particularly crucial for augmenting training and exercise results. Domestic and foreign researchers have recently conducted several studies on antifatigue active substances in animals and plants. They found that some natural active ingredients, such as polypeptides, amino acids, polysaccharides, vitamins, and polyphenols can exert certain antifatigue effects (Zhou et al., 2023). These substances exist widely in food-borne animals and plants. Active substances with antioxidant or anti-inflammatory properties, such as curcumin (McFarlin et al., 2016), omega-3 fatty acids (Lewis et al., 2015), and polyphenolic extracts (Cases et al., 2017), are currently popular sports nutrition supplements. Attention has recently been focused on the relationship between gut microbes and exercise performance (Przewłócka et al., 2020). Interfering with the gut microbiome of athletes has certain positive effects, including improved immunity, gastrointestinal health, exercise performance, antioxidant capacity, fatigue recovery, energy supply, nutrient absorption, and body composition. All of these parameters are critical for the endurance of health and performance in athletes (Pyne et al., 2015). Probiotics are an effective means of regulating the composition and function of gut flora. This promotes microbial diversity, increases reproduction, and helps to promote the growth of healthy species. Thus, appropriate doses of probiotic supplementation improve fatigue-related symptoms, reduce exercise fatigue, and accelerate recovery from muscle injuries (Jäger et al., 2019; Li et al., 2022; Yeh et al., 2022; Miray et al., 2024).

The World Health Organization defines probiotics as “living microorganisms that have health benefits when used in sufficient quantities” (Hill et al., 2014; Lee et al., 2022). Specific genera, such as *Lactobacillus* and *Bifidobacterium*, as well as specific species, such as *Lactobacillus rhamnosus*, can improve the gastrointestinal discomfort caused by a single bout of high-intensity or endurance exercise (Zhang et al., 2023). According to Zhang et al., *Lactobacillus casei Zhang* effectively improved the average running time of rats by affecting their intestinal flora and serum metabolism (Li et al., 2022). *Bifidobacterium longum* OLP-01 (OLP-01), isolated from the intestinal microbiome of a female Olympic champion weightlifter, augmented the grip strength and endurance of mouse forelimbs and significantly reduced the serum levels of LA, ammonia, and CK following acute exercise. At the same time, OLP-01 intervention also significantly increased the basic glycogen level in the liver and muscle (Huang et al., 2020). *Lactobacillus paracasei* PS23, isolated from the fecal samples of healthy people, improved muscle mass and strength in aging mice. Additionally, continuous PS23 supplementation for 6 weeks prevented post-muscle injury strength loss and improved blood muscle injury and inflammatory marker levels in young people (Lee et al., 2022). Probiotic supplementation increases energy supply during exercise, which may offer metabolic benefits for athletes during high-intensity exercise and recovery. *L. plantarum* PS128 supplemented during training significantly increased plasma levels of branched-chain amino acids (increased 24–69%) in triathletes compared to the placebo (Yu and Fu, 2023). On isolating a large number of *Leptococcus bacteria* from the fecal samples of elite marathon runners and transplanting them into mice, Sheiman’s team at Harvard University found that the endurance performance of normal mice significantly improved after supplementation. Compared to the control group, the time spent exercising to exhaustion on the treadmill increased by 13% (Scheiman et al., 2019). *L. rhamnosus* exerts substantial antioxidant effects, which can slow down the oxidative damage caused by free radicals generated during exercise, reduce apoptosis, and enhance exercise capacity. Martarelli et al. (2011) found that *L. paracasei* IMC502 and *L. rhamnosus* IMC501 (1 × 10^9^ colony-forming units (CFUs)/days) supplemented for 4 weeks reduced the serum CK level of cyclists after strenuous exercise, promoted fatigue elimination, and improved the test performance of athletes. However, the effects of this LA bacteria on recovery after long-term exercise have been less studied.

In this study, a strain of *L. rhamnosus* SDSP202418 with high gastric acid and bile salt resistance was isolated from traditional shrimp paste to explore the antifatigue effect of this bacterium. This study offers a theoretical basis for developing functional foods that can enhance endurance and relieve fatigue.

## 2 Materials and methods

### 2.1 Sample preparation

*L. rhamnosus* SDSP202418 (SDSP18) was selected and isolated from the shrimp paste, Shandong, China, with the accession number CGMCC No. 29613, and was deposited in the China General Microbiological Culture Collection Center (CGMCC, Beijing, China). Furthermore, the strain was further identified by the BGI Group (Wuhan, China), an independent third party. The strain maintained at −80°C was resuscitated and cultured in MRS. The bacteria were then diluted with 0.9% normal saline to 1.0 × 10^9^ CFU.

### 2.2 Animals studied and treatments administered

Forty Kunming mice (6 weeks old) with weights between 20 and 22 g were obtained from the experimental animal center of the Chongqing Medical University and used in the experiment. The mice were kept in a warm room at 20–24°C, a relative humidity of 50%, and a 12 h/12 h light–dark cycle. All mice received a standard diet, and food and water were freely available to them. The animals were randomized into four groups of six mice each. In the normal (Nor) and control (Con) groups, the mice were treated with 200 μL of 0.9% normal saline. The vitamin C group and the SDSP18 group were treated with 200 mg/kg of vitamin C and SDSP18 (1.0 × 10^9^ CFU/mL), respectively. The experimental protocol was approved by the Ethics Committee of Chongqing Collaborative Innovation Center for Functional Food (2023102702B, Chongqing, China).

### 2.3 Treadmill fatigue test

**Figure 1:**
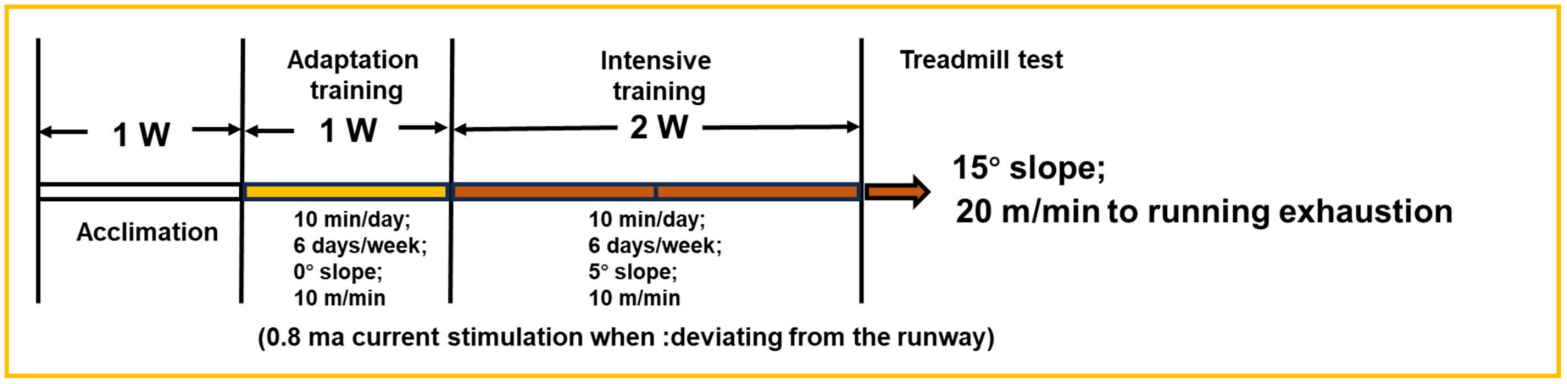
Experiment scheme of Kunming mice.

After 1 week of intragastric administration, the 3-week training began. A treadmill (YH-CS, Wuhan Yihong Technology Co., Ltd., Wuhan, Hubei, China) was used to train the mice after 30 min of gavage. The treadmill running protocol was designed based on the scheme of Xu et al. (2013) and Okamoto et al. (2019) as shown in Figure 1 with some modifications. The normal group was not subjected to any exercise. The other groups of mice underwent 1 week of adaptive training, followed by 2 weeks of intensive training. The adaptation training was established as follows: the speed was 10 m/min, the slope was 0, 10 min a day, 6 consecutive days a week. Intensive training was established at a speed of 10 m/min and a 5° slope for 10 min a day for 6 consecutive days a week. To prevent the mice from deviating from the track, 0.8 ma of electric current was supplied. After 3 weeks, exhaustion tests were conducted on the second day following high-intensity training. The test slope was 15°, running at a speed of 20 m/min. Under electrical stimulation, the mice were considered exhausted when they stopped moving and left the runway for 10 s. Finally, blood was collected from the mouse eyeballs, and the liver, kidney, heart, spleen, lung, and skeletal muscle were dissected for the subsequent analysis (Li et al., 2022).

### 2.4 Physical and chemical indices

The blood was centrifuged at 3,500 rpm for 10 min at 4°C to separate serum, and the serum was then stored at −80°C. Commercial kits (Nanjing Jiancheng Bioengineering Institute, Nanjing, China) were used to determine serum indicators according to instructions.

### 2.5 Hematoxylin and eosin staining

The liver and skeletal muscles of each group were immobilized with 4% polyformaldehyde. The slices were prepared and finally stained with 1% water-soluble eosin staining solution for 5 min. Magnified microscopes (BX43F, Olympus Co., Tokyo, Japan) were used for observing the tissues (200×) after they were sealed with neutral glue.

### 2.6 Real-time PCR

RNA from the liver and muscle was extracted using the TRIzol reagent (Gan et al., 2021). cDNA was synthesized using HiScript SuperMix (Vazyme, Nanjing, China). Real-time quantitative PCR was performed using the StepOnePlus real-time PCR system (Thermo Fisher Scientific, Waltham, MA, United States) and a fluorescent dye (SYBR Green PCR Master Mix). Table 1 presents the primer pairs applied. The target gene expression levels were analyzed using the 2^−ΔΔCT^ method. Beta-actin was selected as an internal parameter (Li et al., 2018).

**Table 1:** Details regarding the primers utilized in real-time PCR.

Gene	Primer sequence (5′ to 3′)
Cu/Zn SOD	Forward: AACCAGTTGTGTTGTCAGGAC Reverse: CCACCATGTTTCTTAGAGTGAGG
Mn SOD	Forward: AGACCTGCCTTACGACTATGG Reverse: CTCGGTGGCGTTGAGATTGTT
CAT	Forward: TGGCACACTTTGACAGAGAGC Reverse: CCTTTGCCTTGGAGTATCTGG
MyHC I	Forward: ACTGTCAACACTAAGAGGGTCA Reverse: TTGGATGATTTGATCTTCCAGGG
MyHC IIa	Forward: TAAACGCAAGTGCCATTCCTG Reverse: GGGTCCGGGTAATAAGCTGG
MyHC IIx	Forward: GCGAATCGAGGCTCAGAACAA Reverse: GTAGTTCCGCCTTCGGTCTTG
MyHC IIb	Forward: CTTTGCTTACGTCAGTCAAGGT Reverse: AGCGCCTGTGAGCTTGTAAA
Sirt 1	Forward: CGCTGTGGCAGATTGTTATTAA Reverse: TTGATCTGAAGTCAGGAATCCC
PGC-1α	Forward: GGATATACTTTACGCAGGTCGA Reverse: CGTCTGAGTTGGTATCTAGGTC

### 2.7 Assays for intestinal microbiota

**Figure 2:**
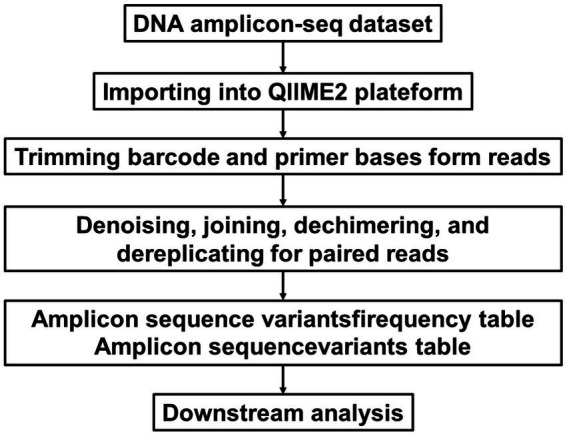
Pre-processing of sequencing data.

Feces were collected from each mouse (*n* = 5) and immediately stored at −80°C. Total genomic DNA was extracted using a fecal genomic DNA extraction kit (TianGen) following the manufacturer’s instructions. The quality of the DNA was checked by spectrophotometric analysis (optical density ratio at 260/280 nm) using ultra-microspectrophotometry and agarose gel electrophoresis (Wang et al., 2020). 16S rRNA gene sequencing was performed. Specific primers 515F and 806R amplified the 16S V4 region. Briefly, PCR reactions were performed with 10 ng gDNA under the following conditions: 98°C, 1 min; 30 cycles: 10s at 98°C, 30s at 50°C, 30s at 72°C; and storage at 72°C for 5 min. Library construction was carried out using the NEBNext^®^ Ultra™ II FS DNA PCR-free Library Prep Kit (New England Biolabs). The constructed libraries were quantified with Qubit and Q-PCR, and after passing quality control, PE 250 sequencing was performed on the NovaSeq 6,000 platform. The augmented sequence data are processed using the QIIME2 Comparison Platform1 (Bolyen et al., 2019). The main process is shown in Figure 2. Split each sample data from the raw data based on the barcode sequence and PCR amplification primer sequence. After trimming the barcode and primer sequences, the reads of each sample were assembled using FLASH (version 1.2.11^1^), resulting in the assembled sequences as the Raw Tags. The assembled Raw Tags were subjected to rigorous filtering using fastp software (version 0.23.1) to obtain high-quality clean tags. After the aforementioned processing, the Tags obtained need to undergo the removal of chimeric sequences. The Tags sequences are aligned with species annotation databases (Silva 138.1 annotation database https://www.arb-silva.de for 16S/18S, Unite database https://unite.ut.ee/ for ITS) to detect chimeric sequences and ultimately remove them to obtain the effective tags.

### 2.8 Statistical analyses

All experiments were performed at least three times. The figures were statistically analyzed and visualized by Origin 2021Pro and SPSS software (version 22.0; IBM Corp., Armonk, NY, United States), and comparison among groups was performed using a one-way ANOVA. All data were expressed as means and standard deviations. Statistical significance was set at a *p*-value of <0.05. We used the LEfSe method, which uses the LEfSe to determine the impact of taxa. Microbiotas displaying differential expression between groups were defined as those with an LDA score of >2.5.

## 3 Results

### 3.1 Effects of *L. rhamnosus* SDSP202418 on body weight and blood glucose of exhausted mice

**Figure 3:**
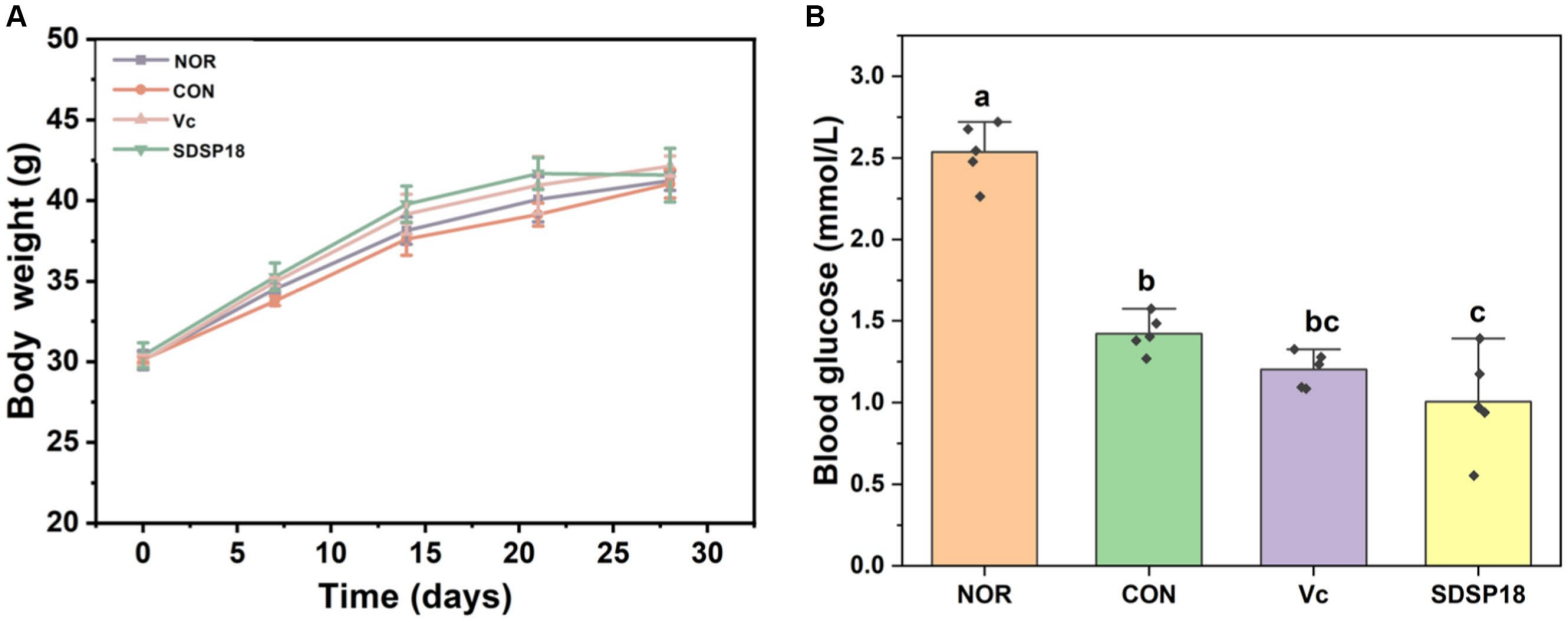
Effect of *Lacticaseibacillus rhamnosus* SDSP202418 on body weight **(A)** and blood glucose **(B)** in mice. The lowercase letters represent significant differences between groups (*p* < 0.05).

No significant difference was observed in the initial body weight of the mice in the four groups (Figure 3A). After SDSP18 was supplemented for 4 consecutive weeks, the body weight of the mice in the four groups increased to varying degrees. The body weights of the control group were 0.4, 2.6, and 1.2% lower than the normal group, vitamin C group, and SDSP18 group, respectively. However, no significant differences were noted (*p* > 0.05). Blood sugar is the substance supplying direct energy to the body during exercise. The blood sugar levels in the exercise mice were significantly lower than those in the normal group (Figure 3B), especially in mice supplemented with vitamin C and probiotics. This may be due to higher glucose consumption in the vitamin C and SDSP18 groups, which means that the SDSP18-supplemented mice receive a more adequate energy supply during exercise, which augments their exercise ability and delays physical fatigue.

### 3.2 Effects of *Lacticaseibacillus rhamnosus* SDSP202118 on exhaustion time and physiological indices of exhausted mice

**Figure 4:**
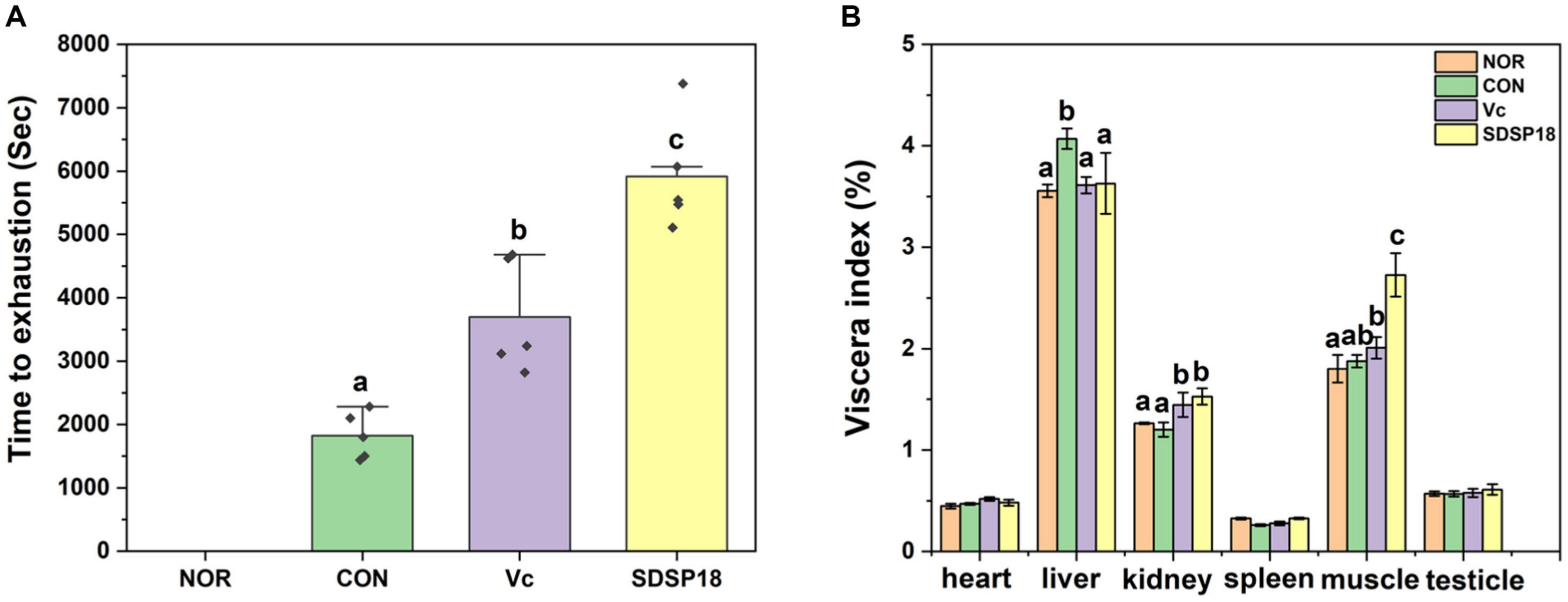
Effect of *Lacticaseibacillus rhamnosus* SDSP202418 on the exhaustive running time **(A)** and body composition **(B)** in mice. The lowercase letters represent significant differences between groups (*p* < 0.05).

Exhaustion time is the most intuitive data representing exercise endurance. No fatigue experiment was conducted in the normal group. After 3 weeks of daily running training, the running exhaustion time of the SDSP18 and vitamin C groups was 3.24 and 2.01 times that of the control group, respectively, with significant differences noted (*p* < 0.05). At the same time, the exhaustion time of the SDSP18 group was 2,219 s longer than that of the vitamin C-positive group (Figure 4A), indicating that SDSP18 supplementation significantly augments endurance and delays fatigue.

Figure 4B presents the ratio of major organs to the body weight of mice. Compared to the normal group, no significant difference was observed in the organ indices of mice in the vitamin C and SDSP18 groups. This indicated that the ingestion of SDSP18 during 3-week running had no effects on the body and organ weights of the mice. The liver index was significantly higher in the exercise group than in the normal group, which indicated that exercise fatigue can cause liver injury. The liver index of the SDSP18 group decreased to the level of the normal group, indicating that SDSP18 exerts a better effect on alleviating the liver injury of mice with exercise fatigue. Compared to the normal group, the muscle index in the other three groups increased, and that in the SDSP18 group significantly increased. This indicated that adaptive exercise improves the muscle index of mice, and probiotic intervention promotes an increase in muscle mass.

### 3.3 Effects of *Lacticaseibacillus rhamnosus* SDSP202418 on biochemical indices of exhausted mice

Table 2 presents the biochemical indices of mice after exhaustive exercise. When the exercise intensity exceeds the aerobic metabolic capacity of the body, LA is produced. This LA accumulates in the body, acidifies muscles, and causes fatigue. The LA content was higher in the vitamin C and SDSP18 groups than in the control group, which may be due to the increased exercise ability of the mice supplemented with antioxidants or probiotics, which in turn increased LA production. BUN is the product of protein aerobic metabolism. If the body’s ability to adapt to exercise load is reduced, the increase in serum BUN levels is more obvious. The serum BUN content in the vitamin C and SDSP18 groups decreased by 32.24 and 41.49% compared to that in the control group, respectively. Lactate dehydrogenase (LDH) primarily catalyzes the reversible reaction between pyruvate and LA. It is a crucial enzyme involved in sugar anaerobic metabolism in the body. Compared to the normal group, LDH levels significantly increased in the control group. Compared to the control group, serum LDH levels in the vitamin C and SDSP18 groups significantly reduced by 24.6 and 42.7%, respectively. The aforementioned indices comprehensively indicated that SDSP18 alleviates exercise fatigue.

**Table 2:** Effect of *Lactobacillus fermentum* SDSP202418 on the serum biochemical parameters of exercise-induced fatigue mice.

	Nor	Con	Vitamin C	SDSP18
LA (μmol/L)	924.42 ± 16.67^a^	945.19 ± 17.49^a^	1012.95 ± 53.30^b^	1318.59 ± 63.30^c^
BUN (μg/mL)	4.35 ± 0.48^a^	10.34 ± 0.36^c^	6.81 ± 0.55^b^	6.05 ± 1.99a^b^
UA (μmol/L)	107.13 ± 16.18^b^	157.89 ± 15.20^c^	70.42 ± 4.21^a^	78.13 ± 8.93^a^
LDH (U/L)	34632.84 ± 3574.78^b^	41367.16 ± 565.06^c^	31191.04 ± 3452.38^b^	23695.52 ± 4065.66^a^
TP (g/L)	4.28 ± 0.07^a^	4.19 ± 0.05^b^	4.17 ± 0.03^b^	4.12 ± 0.04^b^
ALB (g/L)	82.33 ± 6.03	81.97 ± 1.81	84.70 ± 2.46	83.66 ± 8.48
AST (U/L)	19.78 ± 1.79^a^	40.34 ± 2.81^c^	26.09 ± 4.44^b^	30.73 ± 4.44^b^
ALT (U/L)	7.53 ± 1.01^b^	13.99 ± 2.20^a^	4.70 ± 1.88^c^	5.81 ± 2.53^bc^

The values are expressed as mean ± standard deviation. The lowercase letters represent significant differences between groups (*p* < 0.05).

The liver index of the control group significantly increased (Figure 2A), which indicates abnormal liver development after exercise fatigue. Aspartate aminotransferase (AST) and alanine aminotransferase (ALT) are commonly used as clinical and experimental crucial indicators that reflect liver function. High AST and ALT levels often indicate liver function impairment. Therefore, serum ALT and AST levels were further detected. Serum ALT and AST contents were significantly higher in the control group than in the normal group. SDSP18 and vitamin C could significantly downregulate serum ALT and AST levels in the exercise fatigue mice. These results indicate that SDSP18 improves abnormal liver function after exercise fatigue in mice.

### 3.4 Effects of *Lacticaseibacillus rhamnosus* SDSP202418 on antioxidant levels in exhausted mice

Physical exercise produces numerous free radicals, whereas moderate exercise activates the antioxidant defense system and eliminates free radicals. However, excessive exercise decreases the ability to scavenge free radicals in the body, resulting in cell damage (Liu et al., 2016). Therefore, the antioxidant factors SOD, GSH, and CAT must be urgently removed (Wen et al., 2013; Ziolkowski et al., 2015). Table 3 presents the effects of *L. rhamnosus* SDSP18 on serum antioxidant factors in mice. T-AOC is the total antioxidant capacity, which directly reflects the body’s antioxidant capacity. SOD, CAT, and GSH are the main antioxidant defense systems of the body. The activities of these enzymes can directly reflect the strength of the antioxidant capacity. Exercise reduced the serum T-AOC compared to the normal group. Compared to the control group, the serum T-AOC in the SDSP18 group increased by 24.3%, which was higher than that in the vitamin C group (7.05%). This indicated that SDSP18 was more effective than the antioxidant vitamin C in improving the T-AOC of the mice fatigued after exercise. Regarding antioxidant enzymes, compared to the control group, the CAT activity in the SDSP18 and positive control groups increased by 81.5 and 93.5%, respectively, and the GSH content exhibited no significant difference. In summary, SDSP18 reduces the degree of cellular oxidative damage and enhances the ability of the body’s antioxidant system.

**Table 3:** Effects of *Lacticaseibacillus rhamnosus* SDSP202418 on serum antioxidant capacity in mice.

	T-AOC (mmol/gprot)	CAT (U/mL)	GSH (μmol/L)
Nor	1.89 ± 0.12	288.79 ± 11.98 ^a^	90.50 ± 2.38
Con	1.56 ± 0.47	92.73 ± 10.74^c^	91.55 ± 1.32
Vitamin C	1.67 ± 0.18	178.59 ± 21.21^b^	91.87 ± 0.44
SDSP18	1.94 ± 0.12	167.07 ± 12.80^b^	91.17 ± 1.38

The values are expressed as mean ± standard deviation. Columns with different letters were significantly different at a *p*-value of < 0.05.

**Figure 5:**
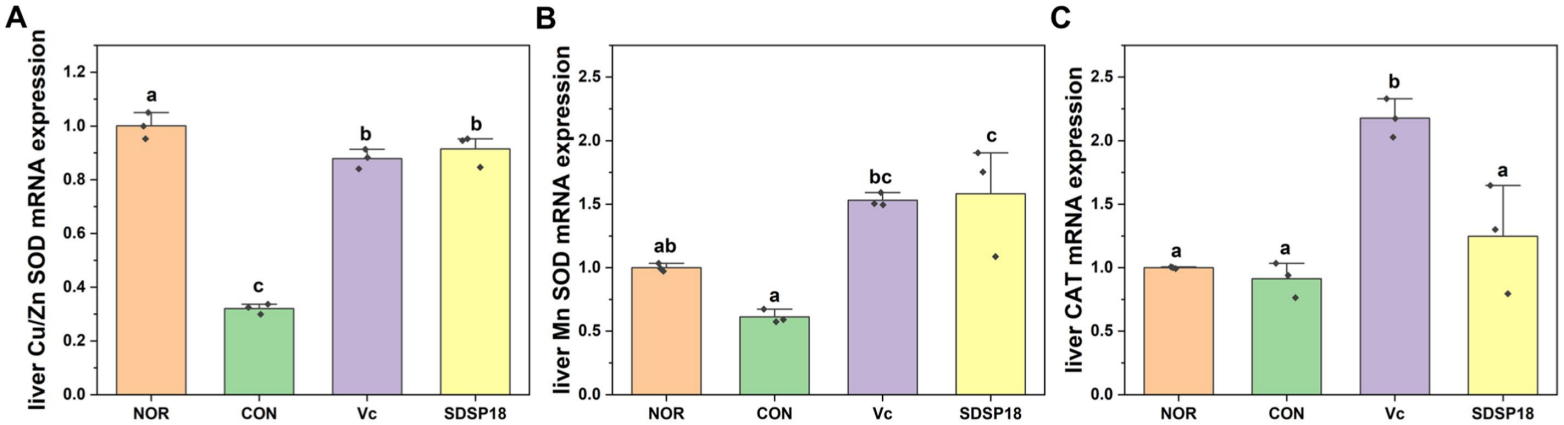
Effects of *Lacticaseibacillus rhamnosus* SDSP202418 on liver antioxidant capacity in mice. The lowercase letters represent significant differences between groups (*p* < 0.05). **(A)** Cu/Zn-SOD mRNA expression; **(B)** Mn-SOD mRNA expression; **(C)** CAT mRNA expression.

Figure 5 presents the mRNA expression of liver oxidative stress-related indicators. Compared to the control group, the mRNA expression levels of Cu/Zn SOD and Mn SOD significantly increased in the SDSP18 group by 185.5 and 158.2%, respectively, and returned to a level equivalent to that in the normal group. These results indicate that SDSP18 improves the body’s antioxidant capacity and relieves fatigue by increasing antioxidant enzyme expression in the liver.

**Figure 6:**
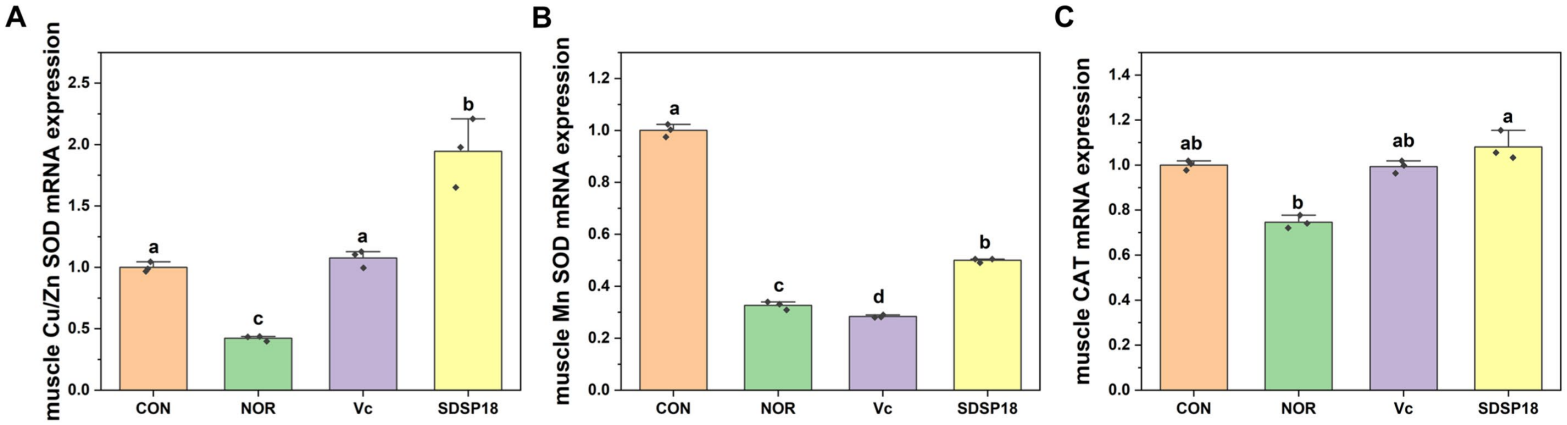
Effects of *Lacticaseibacillus rhamnosus* SDSP18 on muscle antioxidant capacity in mice. The lowercase letters represent significant differences between groups (*p* < 0.05). **(A)** Cu/Zn-SOD mRNA expression; **(B)** Mn-SOD mRNA expression; **(C)** CAT mRNA expression.

Figure 6 presents the mRNA expression levels of Cu/Zn SOD, Mn-SOD, and CAT in the skeletal muscle. SDSP18 significantly promoted the expression of the antioxidant enzyme gene, which increased the Cu/Zn SOD, Mn-SOD, and CAT contents by 362, 53.06, and 65.2%, respectively, compared to the control. At the same time, the effect of increased antioxidase-related genes in the SDSP18 group was better than that in the vitamin C group. These results suggest that, in the skeletal muscle, SDSP18 regulates the expression of oxidative stress-related genes and ameliorates exercise-induced oxidative damage.

### 3.5 *Lacticaseibacillus rhamnosus* SDSP202418 regulates muscle fiber type conversion

**Figure 7:**
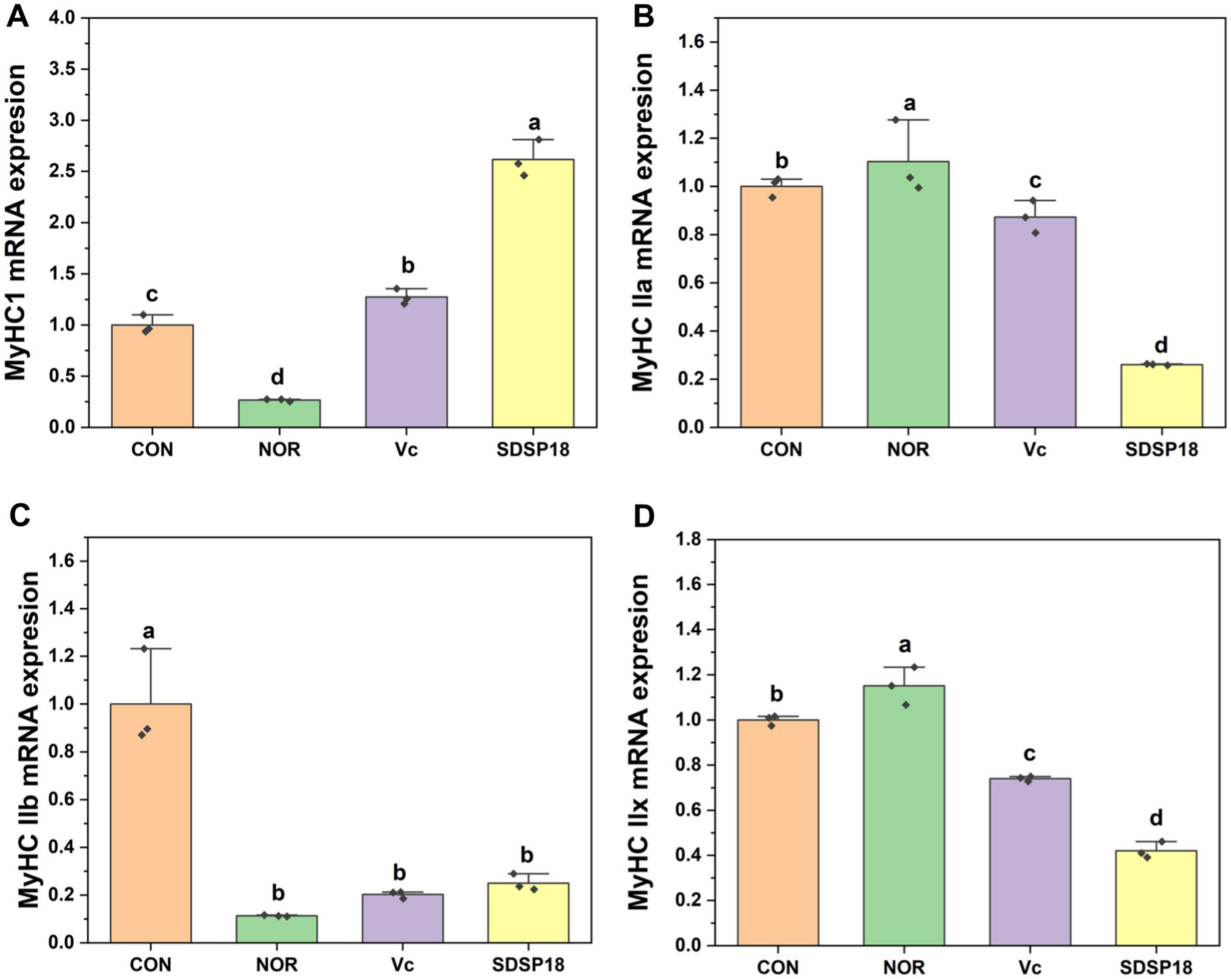
Effect of *Lacticaseibacillus rhamnosus* SDSP202418 on the mRNA expression level of MyHC I **(A)**, MyHC IIa **(B)**, MyHC IIb **(C)**, and MyHC IIx **(D)** in the muscle. The lowercase letters represent significant differences between groups (*p* < 0.05).

The composition of different muscle fibers has a crucial regulatory role in muscle function and exercise endurance. Vitamin C and SDSP18 significantly increased the expression of slow muscle fibers (MyHC I) 6 times and 13 times that of the control (Figure 7). Meanwhile, SDSP18 significantly downregulated the expression of the three fast muscle fibers (MyHC IIa, IIb, and IIx).

**Figure 8:**
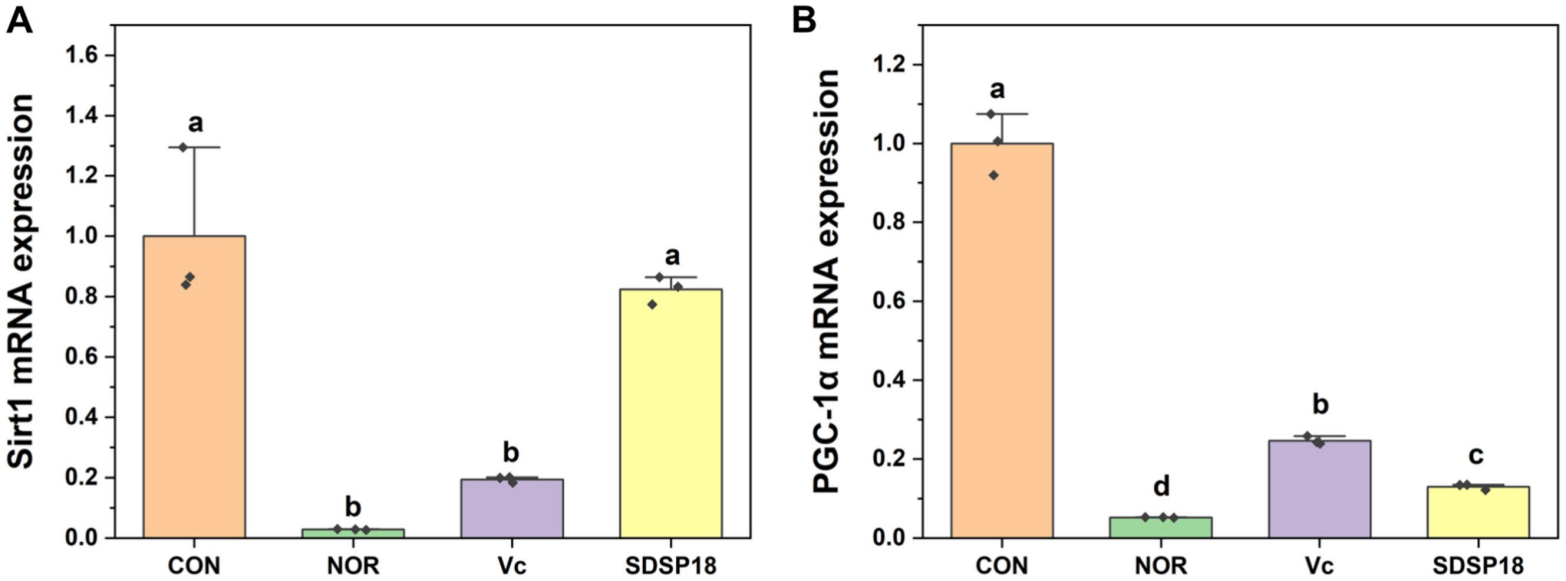
Effect of *Lacticaseibacillus rhamnosus* SDSP202118 on the mRNA expression level of PGC-1α **(A)** and Sirt1 **(B)** in the muscle. The lowercase letters represent significant differences between groups (*p* < 0.05).

Figure 8 presents the mRNA expression levels of the AMPK/peroxisome proliferator-activated receptor gamma coactivator 1α (PGC-1α) pathway-related genes. Compared to the control group, the SDSP18 group exhibited significantly upregulated expression of PGC-1α and sirtuin1 (Sirt1) genes. The expression increased by 1.6 and 20 times, respectively. Of note, SDSP18 had a considerably higher gene expression-promoting effect than the positive control.

### 3.6 Effects of *Lacticaseibacillus rhamnosus* SDSP202418 on histology

**Figure 9:**
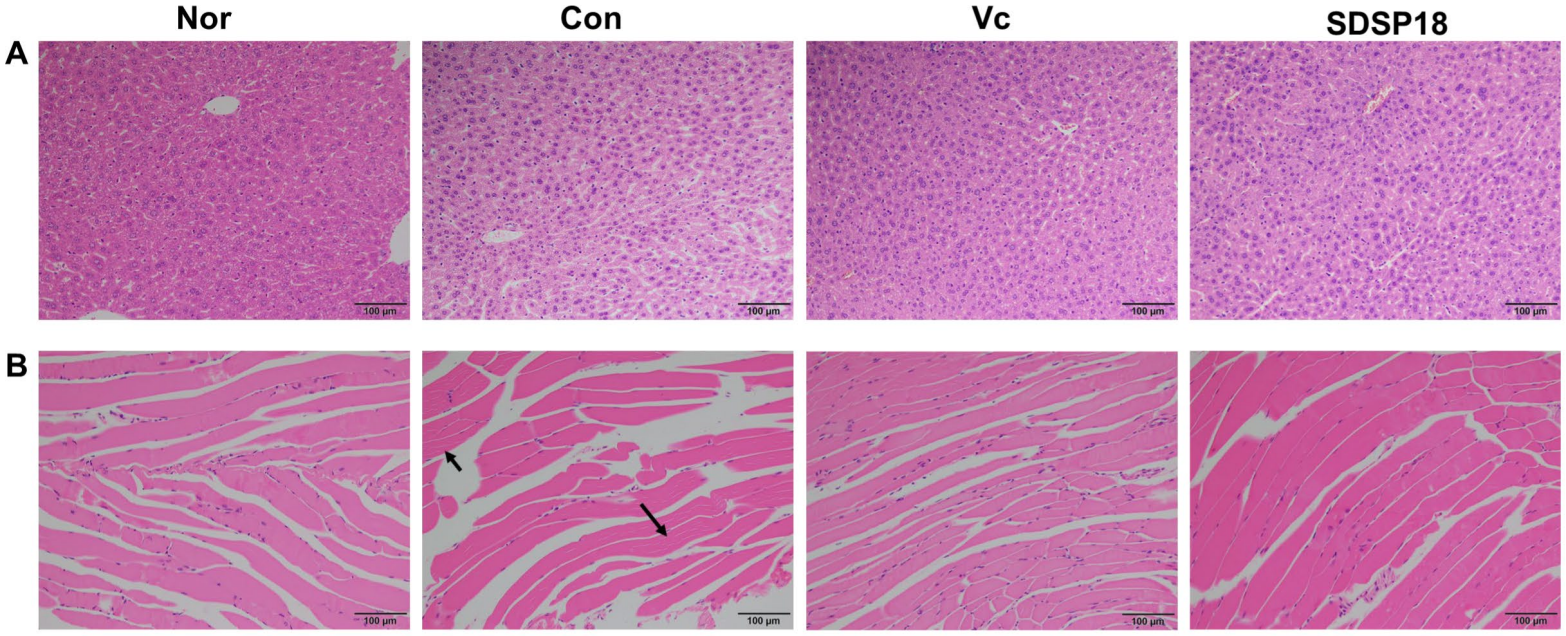
Effects of exercise and vitamin C or SDSP18 interventions on histopathology. **(A)** Liver and **(B)** skeletal muscle. Specimens are photographed using a light microscope (200×).

Figure 9 presents the evaluation results of potential pathological changes in different liver and muscle tissues following programmed exercise training and probiotic supplementation. In the normal group, the liver cells were arranged radially from the central vein, and sinusoid and hepatic cords were arranged in order. By contrast, in the control group, which underwent forced exercise, the liver cells exhibited a disordered arrangement, and the nuclei and cytoplasm were condensed with broken nuclear membranes and nucleoli. The arrangement of sinusoid and hepatic cords exhibited no significant change among the groups after the mice were supplemented with vitamin C or SDSP18 (Figure 9A), similar to those in the normal group. This indicated that SDSP18 supplementation improves the oxidative damage of the mouse liver tissue.

Over-exercise can cause muscle tissue damage, which is manifested by blurred boundaries of muscle bundles, irregular arrangement of muscle fibers, interlaminar spreading, increased interstitial space, connective tissue hyperplasia, inflammatory cell infiltration, and apoptosis (Yu et al., 2022). No significant changes in muscle tissue were seen in the normal group. The control group showed large tissue gaps, probably caused by muscle strain. Dietary supplementation with SDSP18 or vitamin C improved this phenomenon. It also reduced inflammatory infiltration and muscle fiber gaps and prevented exercise-induced muscle tissue damage, thereby improving exercise endurance in mice (Figure 9B).

### 3.7 Effects of *Lacticaseibacillus rhamnosus* SDSP202418 on intestinal microbiota in fatigued mice

**Figure 10:**
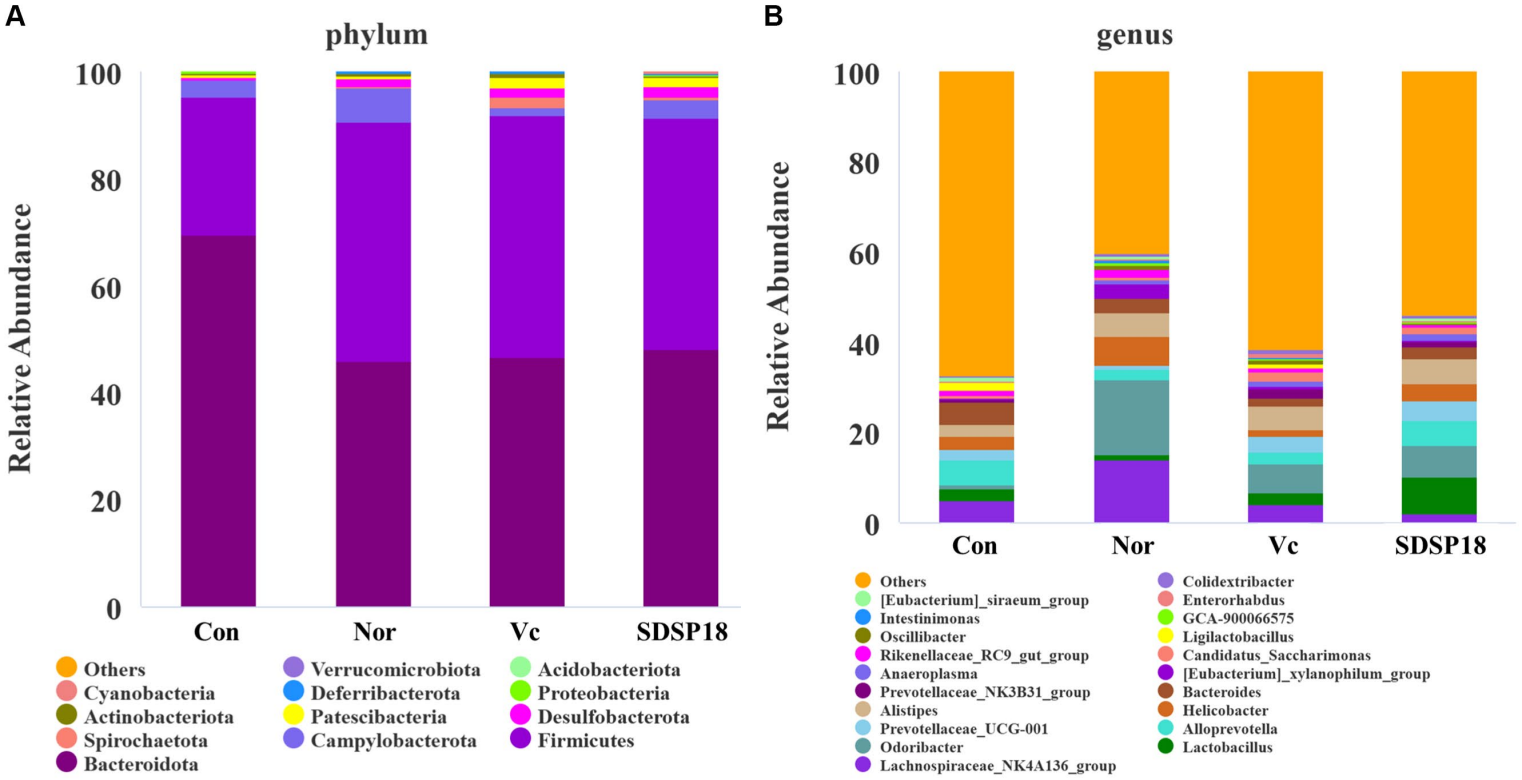
Assay for feces microbiota in mice administered with different treatments. The top 20 abundance from the taxa analysis of microbiota composition at **(A)** phylum and **(B)** genus.

Our results indicated that among the top 20 most abundant microbial phylum and genera, the levels of *Bacteroidota* in fatigued mice were higher than that in fatigued mice treated with vitamin C or SDSP18 (Figure 10A). The species distribution map (Figure 10B) showed that the dominant species at the genus level were *Lactobacillus*, *Odoribacter*, *Prevotellaceae_UCG-001*, *Alistipes*, and *Anaeroplasma* in fatigued mice administered vitamin C or SDSP18. Compared to the normal group, the unclassified and other groups of microorganisms increased in the group control, *Lachnospiraceae_NK4A136_group* decreased, and the populations of *Lactobacillus*, *Odoribacter, Prevotellaceae_UCG-001*, *Alistipes,* and *Anaeroplasma* in the group vitamin C and the group SDSP18 increased, compared to the control group.

**Figure 11:**
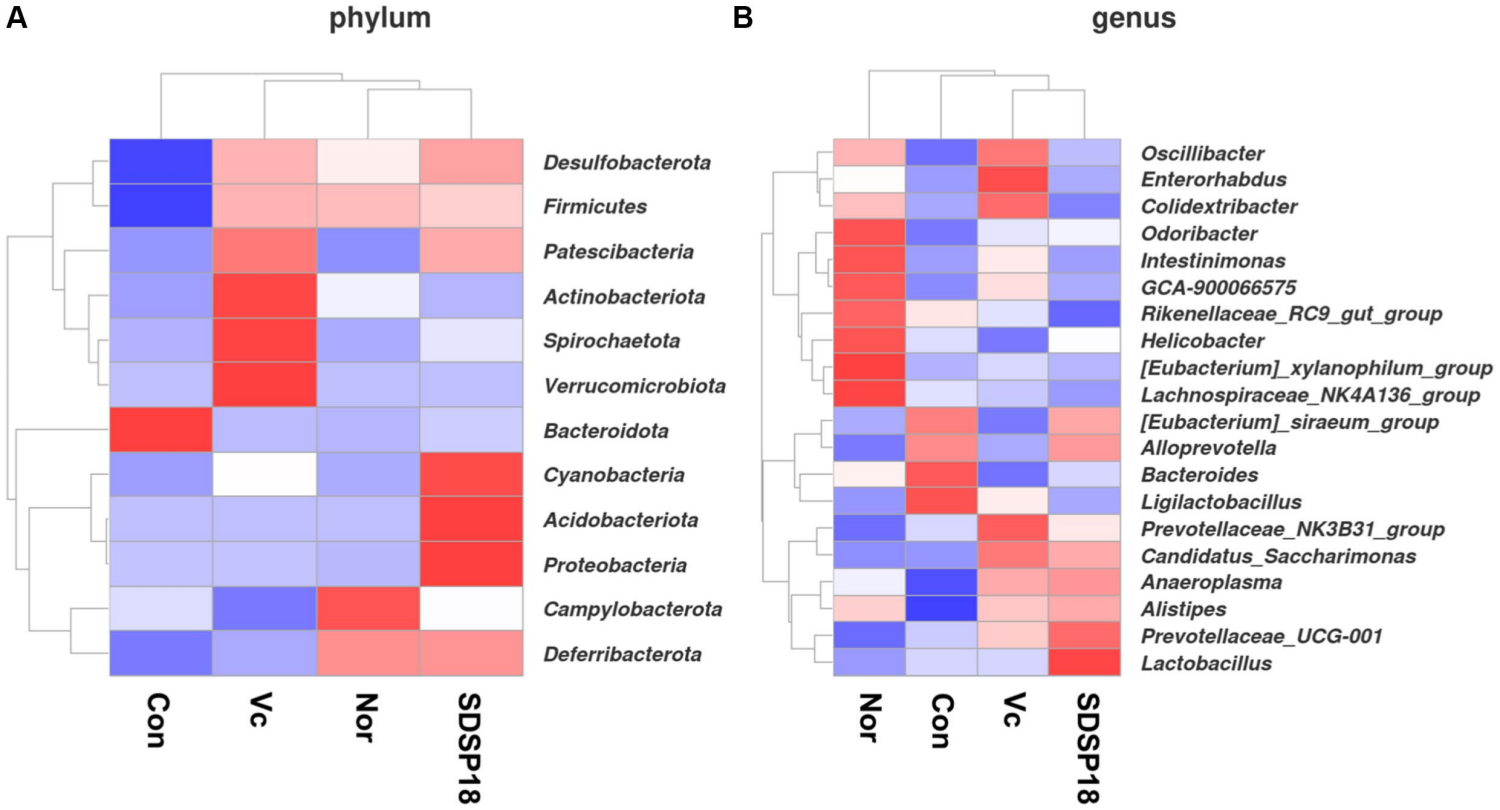
Heat map of species abundance clustering for **(A)** phylum level and **(B)** genus level in mice with different treatments.

We analyzed the gut microbiota at the phylum and genus level using thermography, and Figure 9 shows the proportion of fecal microbiota in mice with different treatments (Figure 11). The intestinal abundance of the *Bacteroidota* group (phylum) elevated in exercised mice. The abundance of *[Eubacterium]_siraeum_group*, and *Ligilactobacillus* was also observed to increase. However, these bacteria were inhibited in fatigued mice given by vitamin C or *Lacticaseibacillus rhamnosus* SDSP18, even though populations of *Alistipes* and *Prevotellaceae_UCG-001* were increased in fatigued mice treated with SDSP18. We also found that the *Lactobacillus* and *Alloprevotella* levels were increased in fatigued mice by administering SDSP18. A similar result that the administration of LA bacteria elevated the abundance of *Bifidobacterium*, *Bacteroides*, *Alistipes*, and *Alloprevotella* has been reported (Li et al., 2020). Meanwhile, exercise and SDSP18 can also decrease the abundance of *Candidatus_Saccharimonas*, compared to the normal group. Our results suggested that SDSP18 elevated the levels of LA bacteria (*Lactobacillus*) and maintained intestinal microbiota (*Coriobacteriales*) in fatigued mice.

**Figure 12:**
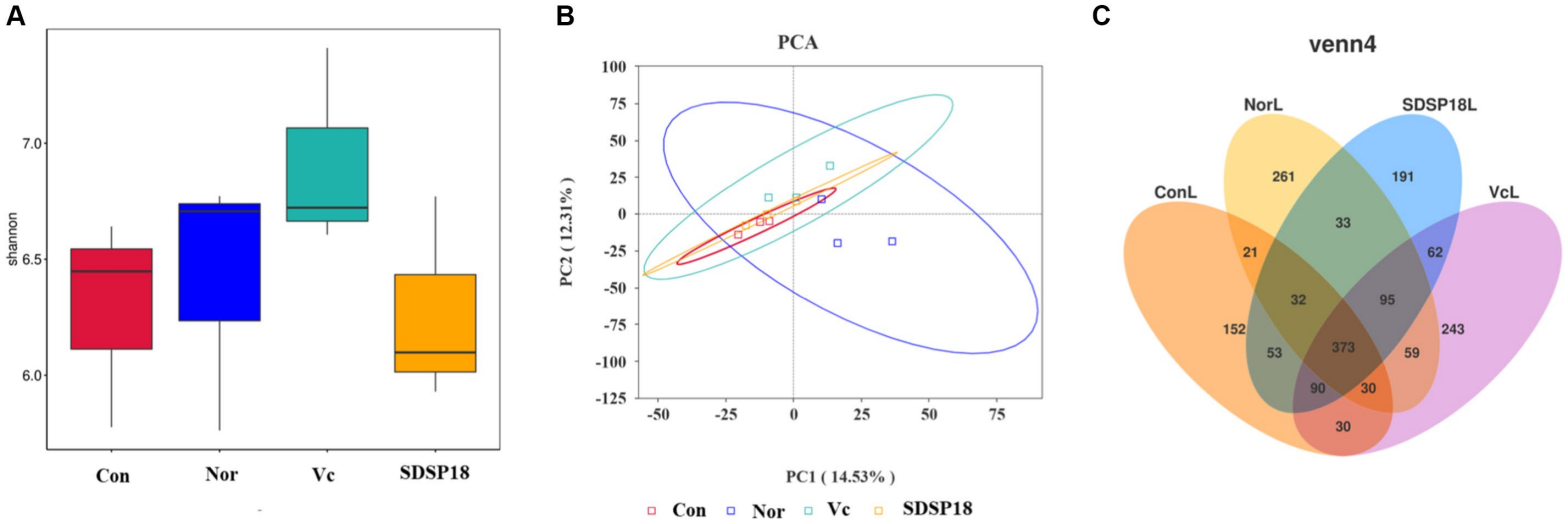
**(A)** Box plot of alpha index distribution between groups and **(B)** genus PCA. **(C)** Flower plot based on OTU abundance.

Changes in the composition of the intestinal flora lead to the formation of dominant species for certain groups of bacteria in the intestinal ecosystem. To assess whether SDSP18 intervention has the potential to modulate intestinal microbiota *in vivo*, this study analyzed mouse fecal bacteria in a 16S metagenomic. The Shannon diversity index was negatively correlated with diversity. No significant change was noted between groups (Figure 12A). Further analysis by PCA also found no obvious differences in the gut microbiota structure between the normal group and other groups (Figure 12B). In addition, 261, 152, 241, and 191 different bacteria (operational taxonomic units; OTUs) were found in normal mice, fatigued mice, and fatigued mice given vitamin C and SDSP18. However, a total of 373 bacterial species were present in these three groups (Figure 12C).

### 3.8 Effects of *Lacticaseibacillus rhamnosus* SDSP202418 on microbial LEfSe analysis

**Figure 13:**
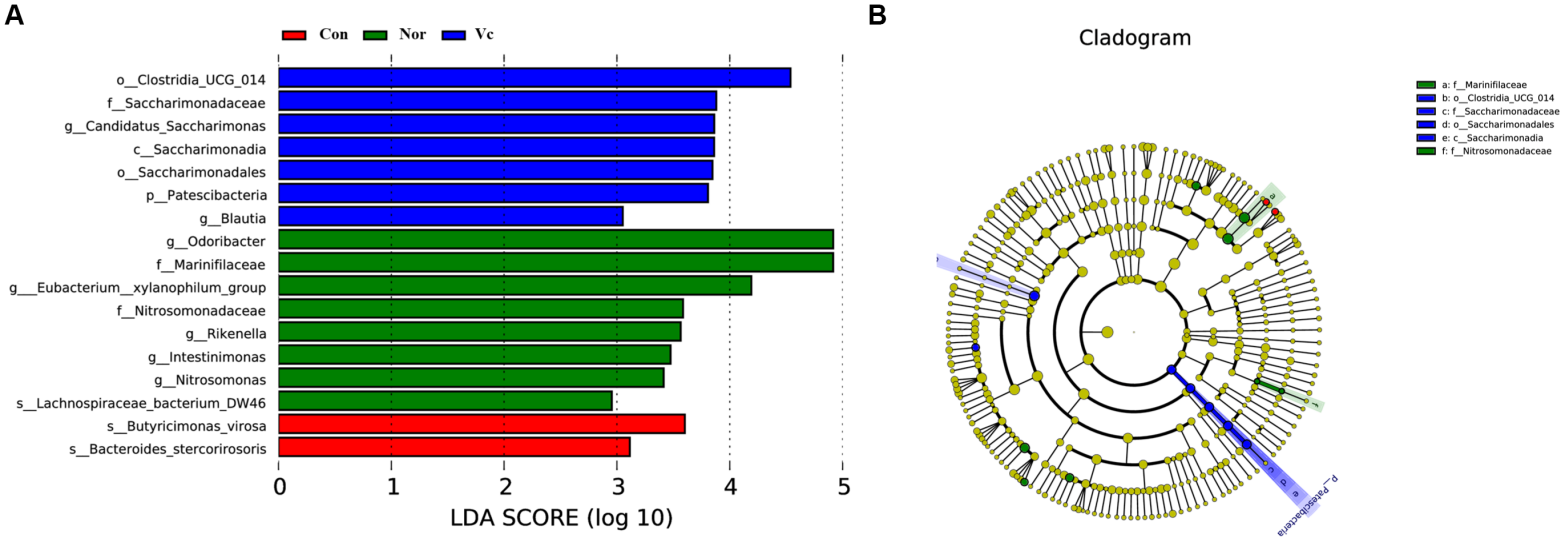
Histogram of the distribution of LDA values **(A)** and evolutionary branching plot **(B)**.

Significantly different abundances between species were revealed by linear discriminant analysis (LDA) and effect size (LEfSe) analysis (Figure 13). Major microbiota, including *Butyricimonas virosa* and *Bacteroides stercorirosoris,* were significantly elevated in fatigued mice and may serve as biomarkers. However, administration of SDSP18 reduced all of these bacteria in the intestines of fatigued mice. We hypothesized that this could be related to the fact that we tested microbes in the feces and that SDSP18 allowed more flora to remain working in the gut. These results suggest that SDSP18 has the potential to modulate the gut microbiota in fatigued mice.

## 4 Discussion

High-intensity exercise leads to metabolite accumulation and consumption of antioxidant enzymes, which results in muscle tension and induces sports injury. The most direct and objective expression of body fatigue is the decline of exercise endurance. Exercise exhaustion time is among the most commonly used indicators of body endurance. In animal experiments, the supplementation of specific probiotics increased the endurance running time of mice to a certain extent (Lee et al., 2020; Scheiman et al., 2019). In the present study, supplementation with *L. rhamnosus* SDSP18 daily during 3 weeks of running training significantly increased the exercise exhaustion time of the mice from 1824 s in the control group to 5,915 s in the SDSP18 group. It was 2,219 s longer than that in the vitamin C-positive group. Supplementation with *L. rhamnosus* SDSP18 for 3 weeks increased muscle index, decreased blood glucose levels after exercise, and improved glucose utilization. This suggested that *L. rhamnosus* SDSP18 may be beneficial for energy supply during exercise, which is consistent with the results of Chen et al. (2016).

LDH is a key enzyme in the body’s glycolysis energy supply system. It reversibly catalyzes pyruvate hydrogenation to LA during the anaerobic glycolysis of sugar. LDH chiefly exists in the skeletal muscle and ensures that muscles can obtain ATP even during a short hypoxia period, which reflects the energy supply characteristics of the glycolysis system. When the tissue is destroyed, LDH from inside the cell enters the blood, which leads to an elevation in serum enzyme activity. In this study, compared to the control group, the supplementation of a certain dose of *L. rhamnosus* SDSP18 significantly reduced the serum LDH level of the exercise mice, indicating that SDSP18 ameliorates exercise-induced cell and muscle injuries to some extent.

LA, urea (UA), and BUN are also biochemical indices of exercise-induced fatigue. UA, a metabolite of protein and amino acid decomposition, is a common indicator used for evaluating the degree of exercise-induced fatigue. Intense exercise and physical labor accelerate nucleotide metabolism, nucleotide, and nucleoside decomposition, and their transformation cycle to produce UA. Accordingly, the blood UA content increases. Therefore, UA is inversely proportional to exercise endurance, that is, the lower the body’s exercise endurance, the more obvious the increased UA content. The experimental results revealed that SDSP18 supplementation restored the UA content in the mice to normal levels after long exhaustive exercise. *L. rhamnosus* SDSP18 improved the energy supply and utilization capacity of the metabolic system and augmented the body’s exercise level. BUN is the key end product of human protein metabolism. When the body exercises for a long period, it is unable to obtain sufficient energy through sugar and fat breakdown. The protein and amino acids in the skeletal muscle participate in catabolism to provide energy and produce BUN. This experiment thus unveiled that SDSP18 supplementation during 3-week running training significantly reduced the serum BUN level in exercise mice. LA is a byproduct of muscles produced during high-intensity exercise when the muscles obtain sufficient energy from the anaerobic glycolysis pathway. Excess LA causes H^+^ and H_3_PO_4_ accumulation in the muscles, thereby decreasing muscle pH, inhibiting phosphofructokinase activity, and reducing the sugar breakdown rate. This ensures that the stromal reticulum binds more Ca^2+^, which then affects the muscle tone. This result is different from those of other studies. In the present study, the serum LA content of the SDSP18-supplemented mice increased, which was speculated to be related to the enhanced tolerance of mice to LA and delayed the emergence of the blood LA fatigue threshold. Combined with exhaustion time, mice in the *L. rhamnosus* SDSP18 group persisted for a long time under the high LA concentration, indicating that probiotic supplementation enhanced the exercise ability of the exercise mice.

Vigorous exercise can accelerate reactive oxygen species (ROS) production, affect the balance of oxygen free radicals and the free radical scavenging system, resulting in oxidative stress damage, and reduce antioxidant enzyme activity (Powers et al., 2004). Growing evidence has shown that ROS induces protein oxidation and strongly promotes muscle fatigue (Yan et al., 2024). Probiotics can reduce oxidative stress, increase exercise capacity, and reduce fatigue levels. CAT and GSH peroxidase (GSH-Px) catalyze H_2_O_2_ decomposition into H_2_O and O_2_, thus protecting the structural and functional integrity of cell membranes. Dietary supplementation with *L. rhamnosus* SDSP18 increased CAT, GSH-Px, and T-AOC activities in the serum of mice with exercise-induced fatigue and reduced oxidative stress-induced damage. SOD is a type of antioxidant metalloenzyme that widely exists in various body tissues and cells. Its main function is to remove excess superoxide anion-free radicals from the body. Both Cu/Zn-SOD and Mn-SOD can inhibit free radicals produced during exercise and are vital for reducing exercise fatigue (Muradian et al., 2002). The study results revealed that *L. rhamnosus* SDSP18 inhibited exercise-induced liver injury by increasing antioxidant enzyme activity in the liver. At the same time, similar results were observed in the muscle tissue, indicating that *L. rhamnosus* SDSP18 protects against fatigue-induced oxidative damage of muscles to a certain extent.

At present, there is no report on the regulation of probiotics on muscle composition. Muscle composition is crucial for resisting exercise fatigue. Therefore, we studied the effect of *L. rhamnosus* SDSP18 on the muscle composition of exhausted mice. Based on contractile and energy metabolic properties, skeletal muscle fibers consist of slow and fast muscle fibers (Chang et al., 2017). Slow muscle fibers (MyHC I) contract at a slower rate, but are able to sustain movement for longer periods of time. Fast muscles contract very fast and are capable of generating strong forces in a short period of time, which can easily lead to fatigue, including MyHC IIx, MyHC IIa, and MyHC IIb (Reyes et al., 2015). SDSP18 can promote the formation of slow muscle fibers and reduce the movement of fast muscle fibers. SDSP18 promotes the formation of slow muscle fibers and reduces the movement of fast muscle fibers. The AMPK/PGC-1α pathway has a crucial role in the regulation of mitochondrial energy metabolism and the regulation of myofiber type (Kahn et al., 2005). GC-1α and Sirt1 are two major downstream factors of AMPK signaling.PGC-1α is closely related to cellular energy metabolism, which is closely related and can activate the expression of slow oxidative fiber genes (Zhang et al., 2017). At the same time, its overexpression increases the proportion of MyHC I (Li et al., 2002). PGC-1α, as a transcriptional coactivator, is closely related to cellular energy metabolism (Lantier et al., 2014). *L. rhamnosus* SDSP18 mitigated exercise-induced downregulation of PGC-1α and Sirt1 gene expression. Meanwhile, SDSP18 promoted the formation of slow muscle fibers and reduced the formation of fast muscle fibers. SDSP18 affects myotube differentiation through the AMPK/PGC-1α pathway and regulates skeletal muscle type conversion, thus enhancing skeletal muscle energy metabolism and alleviating fatigue progression.

The gut microbiota has a crucial role in maintaining human health. Meanwhile, the production and elimination of harmful serum metabolites, oxidative stress response, and muscle are all closely related to gut microbiota *Firmicutes*, *Bacteroidetes*, *Proteobacteria*, and *Actinobacteriota* are the major intestinal flora of the body. The composition and function of the gut microbiota functional components are affected and altered by exercise. Meanwhile, the gut microbiota regulates energy metabolism, inflammatory response, stress resistance, and oxidative stress, which further affect exercise regulation. Many dietary supplements have been shown to improve athletic performance and combat physical fatigue by altering the composition of the gut microbiota. For example, salidroside extract may increase the abundance of beneficial microorganisms, mediate the integrity of the gut barrier, and remodel the gut microbiota (Zhu et al., 2022). Probiotics can produce substantial benefits by directly shaping the microbiota. In the present study, we found no significant changes in microbiota diversity (as reflected by the Shannon diversity index) between the different groups, and the alpha diversity index was not statistically significant. This may not be consistent with a “healthier” microbiota with high species richness. However, considering that the purpose of probiotic supplementation is to allow some gut flora to proliferate more than others to promote beneficial metabolites in gut microbial producers, it is not sufficient to alter overall species diversity, and even species diversity may be compromised. Interestingly, the structure of the gut microbiota changed significantly in the control and SDSP18 groups (as shown in PCoA). We further delved into the changes in the composition of the intestinal flora after the gavage of SDSP18. The number of *Lactobacillus* and *Alistipes* increased in SDSP18-gavaged mice. Among them, *Lactobacillus* could positively regulate exercise-induced fatigue. *Alistipes* are closely related to chronic stress and human health and help to alleviate memory impairment in ecological disorders and diseases. Meanwhile, SDSP18 could reduce the number of *Candidatus Saccharimonas*. These results suggest that SDSP18 may modulate gut flora content, especially by suppressing the content of undesirable flora. The regulation effect of SDSP18 on intestinal microbiota is an important means to alleviate exercise fatigue.

In summary, *L. rhamnosus* SDSP18 alleviates muscle fatigue and improves muscle function through the timely elimination of fatigue-produced metabolites, inhibiting oxidative stress and changing muscle fiber composition.

## 5 Conclusion

In conclusion, the present study demonstrated that *L. rhamnosus* SDSP202418 can alleviate exercise-induced fatigue through several targets, including eliminating harmful serum metabolites, reducing oxidative damage, and changing the muscle fiber composition. Additionally, *L. rhamnosus* SDSP202418 could change the gut microbial structure and modulate the abundance and balance of fatigue-related gut microbiota. Increased probiotics in the gut can restore imbalances in the intestinal ecosystem, normalize serum biomarkers, and reduce oxidative stress, thereby relieving fatigue and enhancing exercise function. Although the direct physiological relationship between skeletal muscle and gut is not obvious, the signals generated by the interaction of intestinal microbiota affect the normal operation of various parts of the body, and can also indirectly affect the physiological activities of muscle tissue. *L. rhamnosus* SDSP202418 may ameliorate fatigue and intestinal injury by targeting small intestinal microbiota. The comprehensive analysis indicated that SDSP18 has the potential to be a novel functional food ingredient for preventing and relieving sports fatigue. In the future, further studies are required to continue to disentangle the complex relationships between *L. rhamnosus* SDSP202418, the gut-muscle axis, and exercise-induced fatigue.

## Data Availability

The original contributions presented in the study are included in the article/Supplementary material, further inquiries can be directed to the corresponding author.

## Footnotes

^1^http://ccb.jhu.edu/software/FLASH/

## References

[ref1] Bolyen E. Rideout J. R. Dillon M. R. Bokulich N. A. Abnet C. C. al-Ghalith G. A.. (2019, 2019). Reproducible, interactive, scalable and extensible microbiome data science using QIIME 2. Nat. Biotechnol. 37, 852–857. doi: 10.1038/s41587-019-0209-9, PMID: 31341288 PMC7015180

[ref2] Cases J. Romain C. Marín-Pagán C. Chung L. H. Rubio-Pérez J. M. Laurent C.. (2017). Supplementation with a polyphenol-rich extract, PerfLoad®, improves physical performance during high-intensity exercise: A randomized, double blind, crossover trial. Nutrients 9:421. doi: 10.3390/nu9040421 28441760 PMC5409760

[ref3] Chang W.-T. Chen C.-S. Cheng M.-C. Wu M.-F. Cheng F.-T. Hsu C.-L. (2017). Effects of resveratrol, epigallocatechin gallate, and epicatechin on mitochondrial functions in C2C12 myotubes. J. Funct. Foods 35, 507–512. doi: 10.1016/j.jff.2017.06.020

[ref4] Chen Y.-M. Wei L. Chiu Y.-S. Hsu Y.-J. Tsai T.-Y. Wang M.-F.. (2016). *Lactobacillus plantarum* TWK10 supplementation improves exercise performance and increases muscle mass in mice. Nutrients 8:205. doi: 10.3390/nu8040205, PMID: 27070637 PMC4848674

[ref5] Gan Y. Tong J. Zhou X. R. Long X. Y. Pan Y. N. Liu W. W.. (2021). Hepatoprotective effect of *Lactobacillus plantarum* HFY09 on ethanol-induced liver injury in mice. Front. Nutr. 8:684588. doi: 10.3389/fnut.2021.684588 34249992 PMC8264191

[ref6] Gonzalez J. T. Fuchs C. J. Betts J. A. van Loon L. J. C. (2016). Liver glycogen metabolism during and after prolonged endurance-type exercise. Am. J. Physiol. Endocrinol. Metab. 311, E543–E553. doi: 10.1152/ajpendo.00232.2016, PMID: 27436612

[ref7] Hill C. Guarner F. Reid G. Gibson G. R. Merenstein D. J. Pot B.. (2014). Expert consensus document: The International Scientific Association for Probiotics and Prebiotics consensus statement on the scope and appropriate use of the term probiotic. Nat. Rev. Gastroenterol. Hepatol. 11, 506–514. doi: 10.1038/nrgastro.2014.66 24912386

[ref8] Huang W.-C. Hsu Y.-J. Huang C.-C. Liu H.-C. Lee M.-C. (2020). Exercise training combined with *Bifidobacterium longum* OLP-01 supplementation improves exercise physiological adaption and performance. Nutrients 12:1145. doi: 10.3390/nu12041145, PMID: 32325851 PMC7231274

[ref9] Jäger R. Mohr A. E. Carpenter K. C. Kerksick C. M. Purpura M. Moussa A.. (2019). International society of sports nutrition position stand: probiotics. J. Int. Soc. Sports Nutr. 16, 1–44. doi: 10.1186/s12970-019-0329-0 31864419 PMC6925426

[ref10] Kahn B. B. Alquier T. Carling D. Hardie D. G. (2005). AMP-activated protein kinase: ancient energy gauge provides clues to modern understanding of metabolism. Cell Metab. 1, 15–25. doi: 10.1016/j.cmet.2004.12.003, PMID: 16054041

[ref11] Lantier L. Fentz J. Mounier R. Leclerc J. Treebak J. T. Pehmøller C.. (2014). AMPK controls exercise endurance, mitochondrial oxidative capacity, and skeletal muscle integrity. FASEB J. 28, 3211–3224. doi: 10.1096/fj.14-250449 24652947

[ref12] Lee M.-C. Ho C.-S. Hsu Y.-J. Huang C.-C. (2022). Live and heat-killed probiotic *Lactobacillus paracasei* PS23 accelerated the improvement and recovery of strength and damage biomarkers after exercise-induced muscle damage. Nutrients 14:4563. doi: 10.3390/nu14214563, PMID: 36364825 PMC9658587

[ref13] Lee M.-C. Hsu Y.-J. Ho H.-H. Hsieh S.-H. Kuo Y.-W. Sung H.-C.. (2020). *Lactobacillus salivarius* subspecies salicinius SA-03 is a new probiotic capable of enhancing exercise performance and decreasing fatigue. Microorganisms. 8:545. doi: 10.3390/microorganisms8040545, PMID: 32283729 PMC7232535

[ref14] Lewis E. J. Radonic P. W. Wolever T. M. Wells G. D. (2015). 21 days of mammalian omega-3 fatty acid supplementation improves aspects of neuromuscular function and performance in male athletes compared to olive oil placebo. J. Int. Soc. Sports Nutr. 12:28. doi: 10.1186/s12970-015-0089-4, PMID: 26085822 PMC4470064

[ref15] Li J. Kinoshita T. Pandey S. Ng C. K.-Y. Gygi S. P. Shimazaki K.-I.. (2002). Modulation of an RNA-binding protein by abscisic-acid-activated protein kinase. Nature 418, 793–797. doi: 10.1038/nature00936 12181571

[ref16] Li H. Z. Liu F. Lu J. J. Shi J. L. Guan J. Q. Yan F. F.. (2020). Probiotic mixture of *Lactobacillus plantarum* strains improves lipid metabolism and gut microbiota structure in high fat diet-fed mice. Front. Microbiol. 11. doi: 10.3389/fmicb.2020.00512 PMC7113563 32273874

[ref17] Li Y. Wang S. Quan K. Ma D. Zhang H. Zhang W.. (2022). Co-administering yeast polypeptide and the probiotic, Lacticaseibacillus casei Zhang, significantly improves exercise performance. J. Funct. Foods 95:105161. doi: 10.1016/j.jff.2022.105161

[ref18] Li F. Zhou H. Zhou X. Yi R. Mu J. Zhao X.. (2018). *Lactobacillus plantarum* CQPC05 isolated from pickled vegetables inhibits constipation in mice. Appl. Sci. 9, 159–171. doi: 10.3390/app9010159

[ref20] Liu S. Meng F. Zhang D. Shi D. Zhou J. Guo S.. (2022). *Lonicera caerulea* berry polyphenols extract alleviates exercise fatigue in mice by reducing oxidative stress, inflammation, skeletal muscle cell apoptosis, and by increasing cell proliferation. Front Nutr. 9:853225. doi: 10.3389/fnut.2022.853225, PMID: 35356725 PMC8959458

[ref21] Liu R. Xing L. Fu Q. Zhou G.-H. Zhang W.-G. (2016). A review of antioxidant peptides derived from meat muscle and by-products. Antioxidants. 5:32. doi: 10.3390/antiox5030032, PMID: 27657142 PMC5039581

[ref22] Martarelli D. Verdenelli M. C. Scuri S. Cocchioni M. Silvi S. Cecchini C.. (2011). Effect of a probiotic intake on oxidant and antioxidant parameters in plasma of athletes during intense exercise training. Curr. Microbiol. 62, 1689–1696. doi: 10.1007/s00284-011-9915-3, PMID: 21400082

[ref23] McFarlin B. K. Venable A. S. Henning A. L. Sampson J. N. B. Pennel K. Vingren J. L.. (2016). Reduced inflammatory and muscle damage biomarkers following oral supplementation with bioavailable curcumin. BBA Clin. 5, 72–78. doi: 10.1016/j.bbacli.2016.02.003 27051592 PMC4802396

[ref24] Miray N. A. Esma N. E. Menşure N. Ç. Murat G. (2024). An updated view of the effect of probiotic supplement on sports performance: a detailed review. Curr. Nutr. Rep. 13, 251–263. doi: 10.1007/s13668-024-00527-x 38470560 PMC11133216

[ref25] Muradian K. K. Utko N. A. Fraifeld V. Mozzhukhina T. G. Pishel I. N. Litoshenko A. Y. (2002). Superoxide dismutase, catalase and glutathione peroxidase activities in the liver of young and old mice: linear regression and correlation. Arch. Gerontol. Geriatr. 35, 205–214. doi: 10.1016/S0167-4943(02)00025-0, PMID: 14764359

[ref26] Nikolaidis S. Kosmidis I. Sougioultzis M. Kabasakalis A. Mougios V. (2018). Diurnal variation and reliability of the urine lactate concentration after maximal exercise. Chronobiol. Int. 35, 24–34. doi: 10.1080/07420528.2017.1380037, PMID: 29172728

[ref27] Okamoto T. Morino K. Ugi S. Nakagawa F. Lemecha M. Ida S.. (2019). Microbiome potentiates endurance exercise through intestinal acetate production. Am. J. Physiol. Endocrinol. Metab. 316, E956–E966. doi: 10.1152/ajpendo.00510.2018 30860879

[ref28] Powers S. K. Deruisseau K. C. Quindry J. Hamilton K. L. (2004). Dietary antioxidants and exercise. J. Sports Sci. 22, 81–94. doi: 10.1080/0264041031000140563 14971435

[ref29] Przewłócka K. Folwarski M. Kaźmierczak-Siedlecka K. Skonieczna-Żydecka K. Kaczor J. J. (2020). Gut-muscle axis exists and may affect skeletal muscle adaptation to training. Nutrients 12:1451. doi: 10.3390/nu12051451, PMID: 32443396 PMC7285193

[ref30] Pyne D. B. West N. P. Cox A. J. Cripps A. W. (2015). Probiotics supplementation for athletes–clinical and physiological effects. Eur. J. Sport Sci. 15, 63–72. doi: 10.1080/17461391.2014.971879 25339255

[ref31] Reyes N. L. Banks G. B. Tsang M. Margineantu D. Gu H. Djukovic D.. (2015). Fnip 1 regulates skeletal muscle fiber type specification, fatigue resistance, and susceptibility to muscular dystrophy. Proc. Natl. Acad. Sci. U S A 112, 424–429. doi: 10.1073/pnas.1413021112, PMID: 25548157 PMC4299192

[ref32] Scheiman J. Luber J. M. Chavkin T. A. MacDonald T. Tung A. Pham L.-D.. (2019). Meta-omics analysis of elite athletes identifies a performance-enhancing microbe that functions via lactate metabolism. Nat. Med. 25, 1104–1109. doi: 10.1038/s41591-019-0485-4 31235964 PMC7368972

[ref33] Spector S. A. Jackman M. R. Sabounjian L. A. Sakkas C. Landers D. M. Willis W. T. (1995). Effect of choline supplementation on fatigue in trained cyclists. Med. Sci. Sports Exerc. 27, 668–673, PMID: 7674870

[ref34] Verkerke G. J. Hof A. Zijlstra W. Ament W. Rakhorst G. (2005). Determining the centre of pressure during walking and running using an instrumented treadmill. J. Biomech. 38, 1881–1885. doi: 10.1016/j.jbiomech.2004.08.015, PMID: 16023476

[ref35] Wang J. Bai X. Peng C. Yu Z. Li B. Zhang W.. (2020). Fermented milk containing Lacticaseibacillus casei Zhang and *Bifidobacterium animalis* ssp. Lactis V9 alleviated constipation symptoms through regulation of intestinal microbiota, inflammation, and metabolic pathways. J. Dairy Sci. 103, 11025–11038. doi: 10.3168/jds.2020-18639 33222846

[ref36] Weisleder N. Ma J. (2008). Altered Ca2+ sparks in aging skeletal and cardiac muscle. Ageing Res. Rev. 7, 177–188. doi: 10.1016/j.arr.2007.12.003, PMID: 18272434 PMC2812416

[ref37] Wen K. N. Ching H. W. Teng L. W. Yang H. C. Ching W. K. Mei C. H.. (2013). Hepatoprotective effects of *Ixora parviflora* extract against exhaustive exercise-induced oxidative stress in mice. Molecules. 18, 10721–10732. doi: 10.3390/molecules180910721 24005966 PMC6269953

[ref38] Xu C. Lv J. Lo Y. M. Cui S. W. Hu X. Fan M. (2013). Effects of oat β-glucan on endurance exercise and its anti-fatigue properties in trained rats. Carbohydr. Polym. 92, 1159–1165. doi: 10.1016/j.carbpol.2012.10.023, PMID: 23399141

[ref39] Yan J. N. Zhang Z. J. Zheng J. Li L. Wang C. Lai B.. (2024). The antifatigue effect of scallop male gonad powders via alleviating oxidative stress and modulating inflammatory cytokines in mice. Food Biosci. 59:104259. doi: 10.1016/j.fbio.2024.104259

[ref40] Yeh W.-L. Hsu Y.-J. Ho C.-S. Ho H.-H. Kuo Y.-W. Tsai S.-Y.. (2022). *Lactobacillus plantarum* Pl-02 supplementation combined with resistance training improved muscle mass, force, and exercise performance in mice. Front. Nutr. 9:896503. doi: 10.3389/fnut.2022.896503, PMID: 35571912 PMC9094439

[ref41] Yu C.-H. Fu S.-K. (2023). Effect of *Lactobacillus plantarum* PS128 on neuromuscular efficiency after a half-marathon. Front. Physiol. 14:1254985. doi: 10.3389/fphys.2023.1254985, PMID: 38098805 PMC10720321

[ref42] Yu J. Jiang W. Wang S. Liu S. Shi D. Wang H.. (2022). Microencapsulated hawthorn berry polyphenols alleviate exercise fatigue in mice by regulating AMPK signaling pathway and balancing intestinal microflora. J. Funct. Foods 97:105255. doi: 10.1016/j.jff.2022.105255

[ref43] Zhang Y. Ryu B. Cui Y. Li C. Zhou C. Hong P.. (2019). A peptide isolated from *Hippocampus abdominalis* improves exercise performance and exerts anti-fatigue effects via AMPK/PGC-1α pathway in mice. J. Funct. Foods 61:103489. doi: 10.1016/j.jff.2019.103489

[ref44] Zhang L. Zhang R. Li L. (2023). Effects of probiotic supplementation on exercise and the underlying mechanisms. Food Secur. 12:1787. doi: 10.3390/foods12091787, PMID: 37174325 PMC10178086

[ref45] Zhang L. Zhou Y. Wu W. Hou L. Chen H. Zuo B.. (2017). Skeletal muscle-specific overexpression of PGC-1α induces fiber-type conversion through enhanced mitochondrial respiration and fatty acid oxidation in mice and pigs. Int. J. Biol. Sci. 13, 1152–1162. doi: 10.7150/ijbs.20132, PMID: 29104506 PMC5666330

[ref46] Zhou Y. P. Chu Z. X. Luo Y. Yang F. Y. Cao F. L. Luo F. J.. (2023). Dietary polysaccharides exert anti-fatigue functions via the gut-muscle axis: advances and prospectives. Food Secur. 12:3083. doi: 10.3390/foods12163083, PMID: 37628082 PMC10453516

[ref47] Zhu H. K. Wang R. Y. Hua H. Y. Qian H. Du P. (2022). Deciphering the potential role of maca compounds prescription influencing gut microbiota in the management of exercise-induced fatigue by integrative genomic analysis. Front. Nutr. 9:1004174. doi: 10.3389/fnut.2022.1004174 36313119 PMC9597638

[ref48] Ziolkowski W. Flis D. Halon M. Vadhana D. Olek R. Carloni M.. (2015). Prolonged swimming promotes cellular oxidative stress and p66Shc phosphorylation, but does not induce oxidative stress in mitochondria in the rat heart. Free Radic. Res. 49, 7–16. doi: 10.3109/10715762.2014.968147, PMID: 25287525

