# Supplementary material for: Alleviating effect of Lactobacillus rhamnosus SDSP202418 on exercise-induced fatigue in mice
Source: Front Microbiol. 2024 Sep 26;15:1420872. doi: 10.3389/fmicb.2024.1420872 (PMC11464290; doi:10.3389/fmicb.2024.1420872)
Supplement: Supplementary file 1 [file Data_Sheet_1.ZIP › date/alpha_asv_table_shannon_SDSP8L_tukey.tar.gz!/alpha_asv_table_shannon_SDSP8L_tukey.result/shannon.pdf]

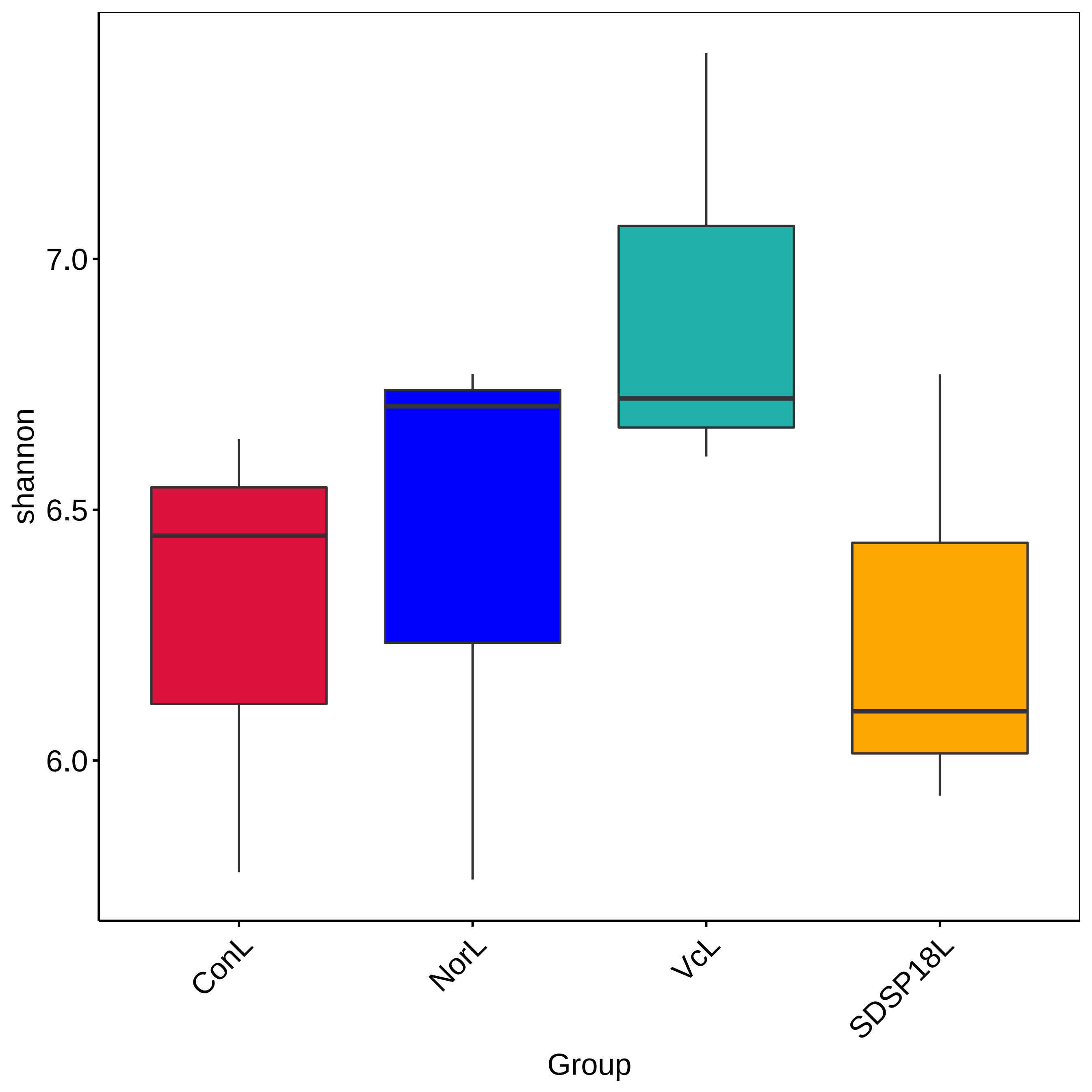

shannon
7.0
6.5
6.0
ConL
NorL
VcL
SDSP18L
Group
