# Supplementary material for: Alleviating effect of Lactobacillus rhamnosus SDSP202418 on exercise-induced fatigue in mice
Source: Front Microbiol. 2024 Sep 26;15:1420872. doi: 10.3389/fmicb.2024.1420872 (PMC11464290; doi:10.3389/fmicb.2024.1420872)
Supplement: Supplementary file 1 [file Data_Sheet_1.ZIP › date/flower_asv_table_SDSP8L_group.1.tar.gz!/flower_asv_table_SDSP8L_group.1.result/flower_asv_table_SDSP8L_group.1.pdf]

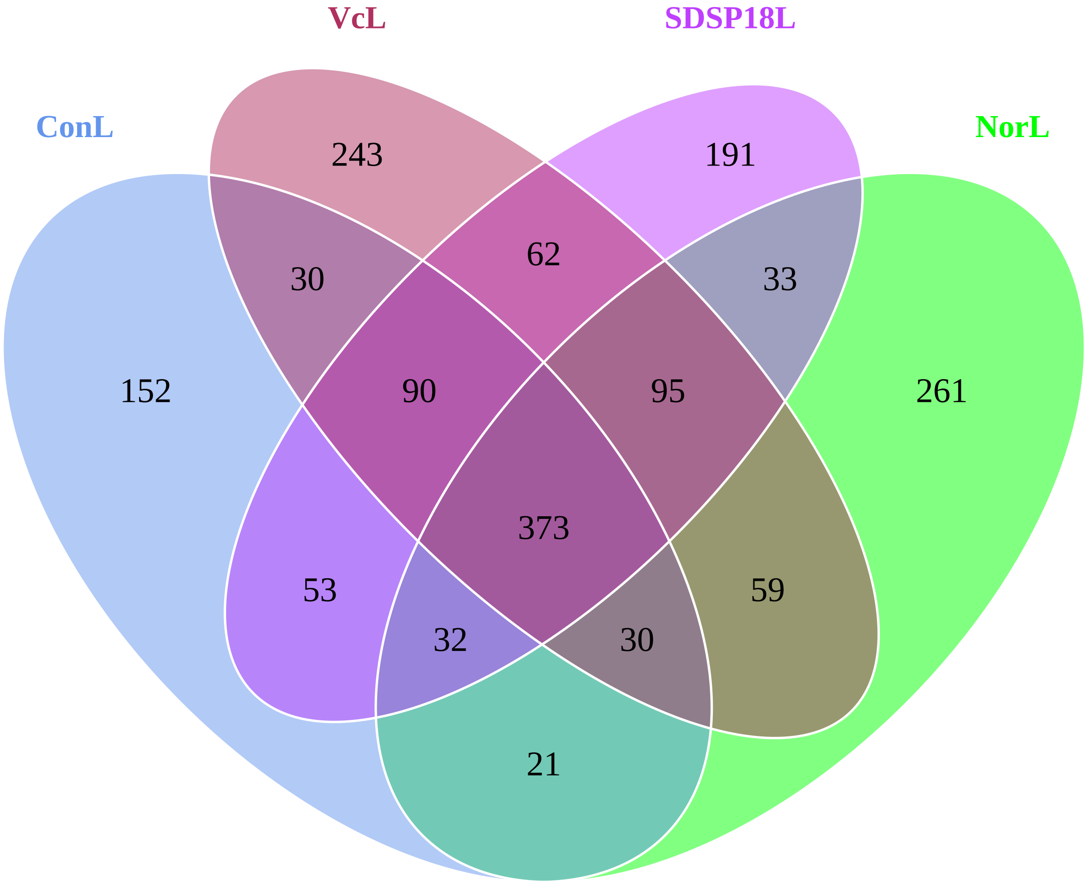

VcL
SDSP18L
ConL
NorL
243
191
62
30
33
152
90
95
261
373
53
59
32
30
21
