# Supplementary material for: Alleviating effect of Lactobacillus rhamnosus SDSP202418 on exercise-induced fatigue in mice
Source: Front Microbiol. 2024 Sep 26;15:1420872. doi: 10.3389/fmicb.2024.1420872 (PMC11464290; doi:10.3389/fmicb.2024.1420872)
Supplement: Supplementary file 1 [file Data_Sheet_1.ZIP › date/heatm_asv_table_SDSP8L_20_phylum,genus,species_group.phylum.tar.gz!/heatm_asv_table_SDSP8L_20_phylum,genus,species_group.phylum.result/heatm_asv_table_SDSP8L_20_phylum,genus,species_group.phylum.pdf]

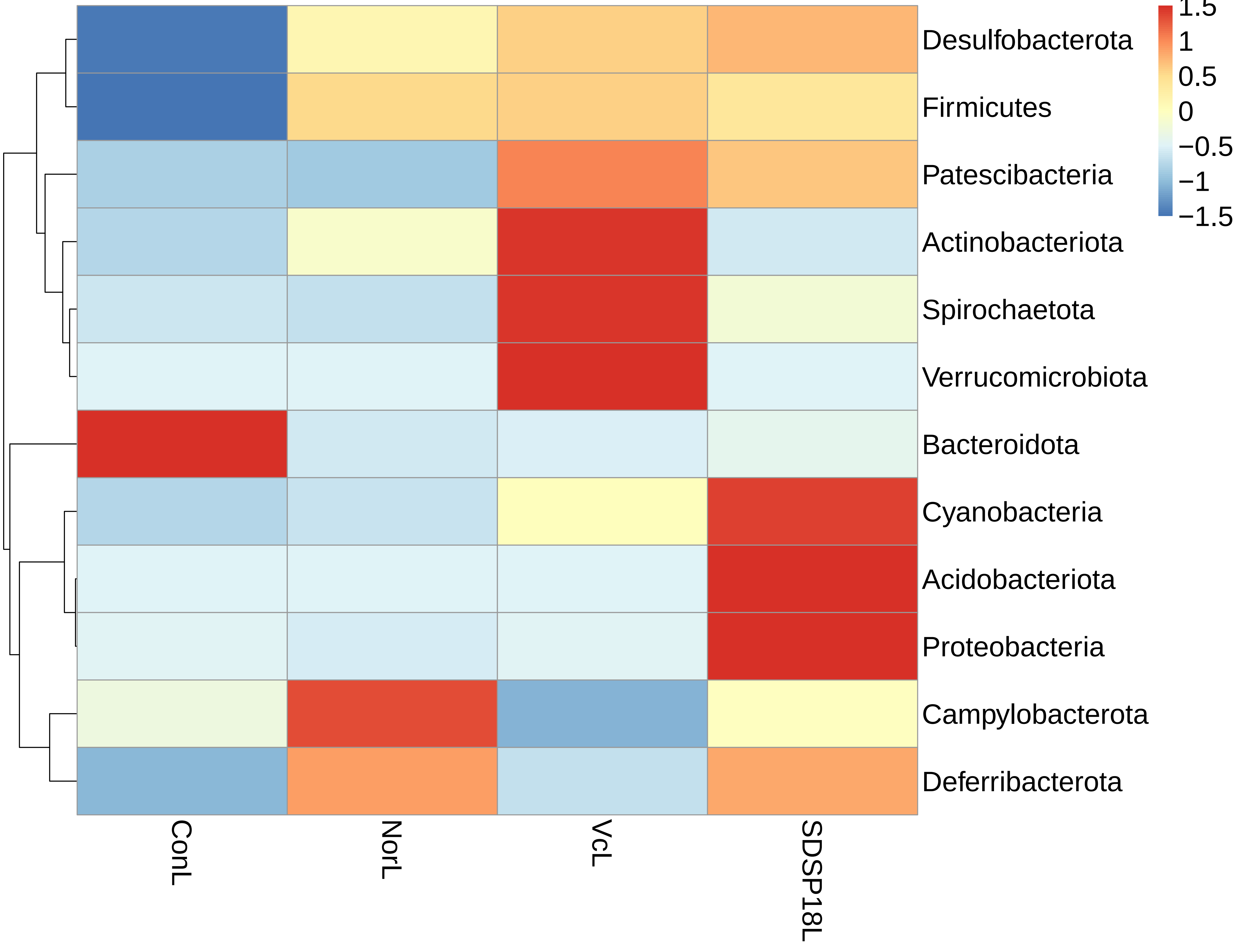

1.5
1
0.5
0
−0.5
−1
−1.5
Desulfobacterota
Firmicutes
Patescibacteria
Actinobacteriota
Spirochaetota
Verrucomicrobiota
Bacteroidota
Cyanobacteria
Acidobacteriota
Proteobacteria
Campylobacterota
Deferribacterota
ConL
NorL
VcL
SDSP18L
