# Supplementary material for: Alleviating effect of Lactobacillus rhamnosus SDSP202418 on exercise-induced fatigue in mice
Source: Front Microbiol. 2024 Sep 26;15:1420872. doi: 10.3389/fmicb.2024.1420872 (PMC11464290; doi:10.3389/fmicb.2024.1420872)
Supplement: Supplementary file 1 [file Data_Sheet_1.ZIP › date/lefse_asv_table_SDSP8L_ConLvsNorLvsVcLvsSDSP18L_2.5.tar.gz!/lefse_asv_table_SDSP8L_ConLvsNorLvsVcLvsSDSP18L_2.5/ConL-vs-NorL-vs-VcL-vs-SDSP18L/LDA.pdf]

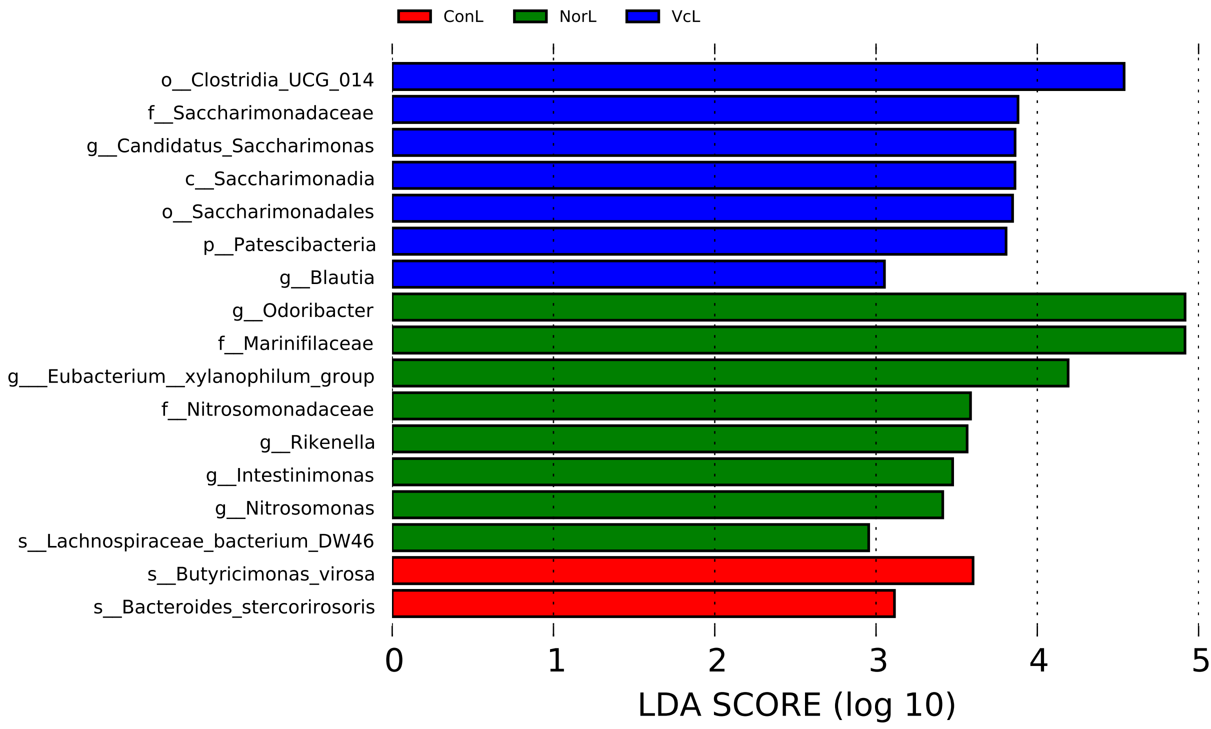
ConL
NorL
VcL
o__Clostridia_UCG_014
f__Saccharimonadaceae
g__Candidatus_Saccharimonas
c__Saccharimonadia
o__Saccharimonadales
p__Patescibacteria
g__Blautia
g__Odoribacter
f__Marinifilaceae
g___Eubacterium__xylanophilum_group
f__Nitrosomonadaceae
g__Rikenella
g__Intestinimonas
g__Nitrosomonas
s__Lachnospiraceae_bacterium_DW46
s__Butyricimonas_virosa
s__Bacteroides_stercorirosoris
0
1
2
3
4
5
LDA SCORE (log 10)
