# Supplementary material for: Alleviating effect of Lactobacillus rhamnosus SDSP202418 on exercise-induced fatigue in mice
Source: Front Microbiol. 2024 Sep 26;15:1420872. doi: 10.3389/fmicb.2024.1420872 (PMC11464290; doi:10.3389/fmicb.2024.1420872)
Supplement: Supplementary file 1 [file Data_Sheet_1.ZIP › date/lefse_asv_table_SDSP8L_ConLvsNorLvsVcLvsSDSP18L_2.5.tar.gz!/lefse_asv_table_SDSP8L_ConLvsNorLvsVcLvsSDSP18L_2.5/ConL-vs-NorL-vs-VcL-vs-SDSP18L/LDA.tree.pdf]

Cladogram

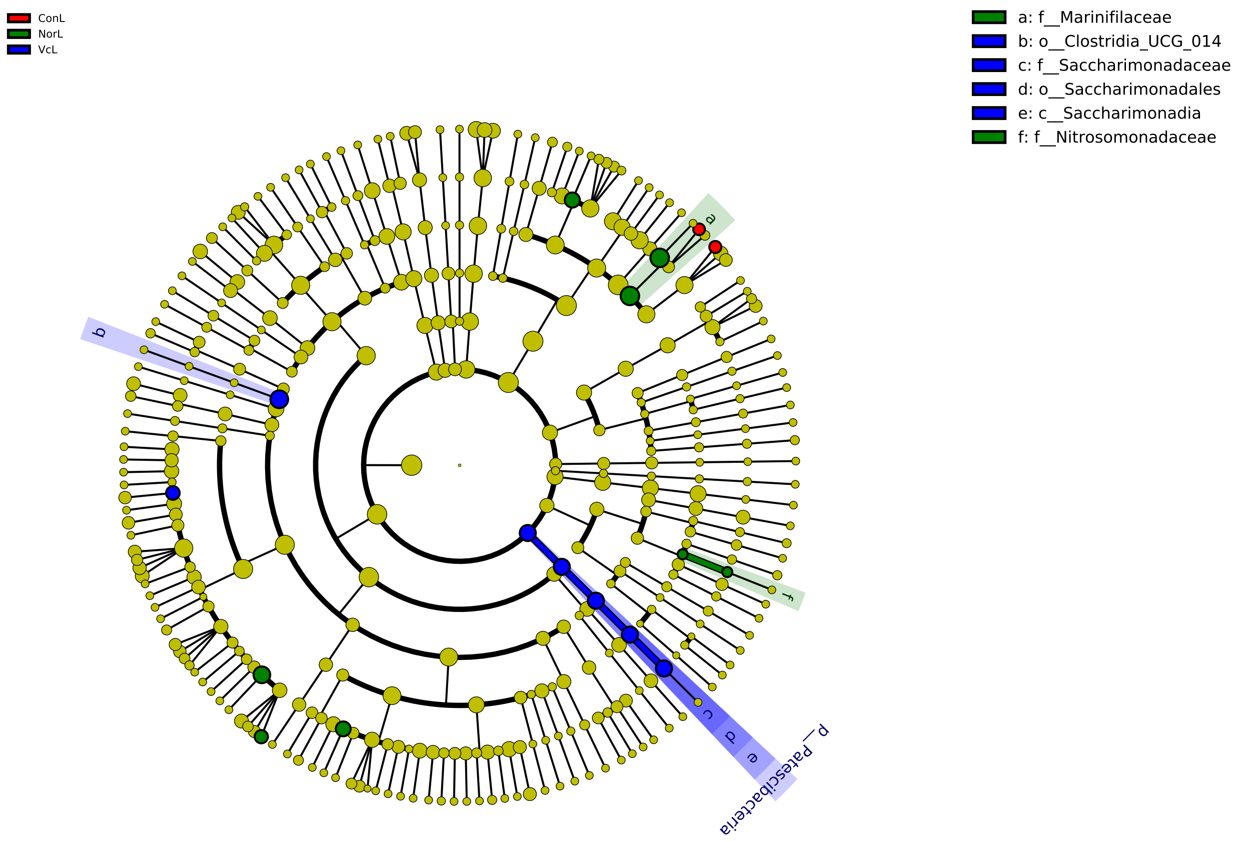

ConL
NorL
VcL
a: f__Marinifilaceae
b: o__Clostridia_UCG_014
c: f__Saccharimonadaceae
d: o__Saccharimonadales
e: c__Saccharimonadia
f: f__Nitrosomonadaceae
a
b
c
d
e
f
p__Patescibacteria
