# Supplementary material for: Alleviating effect of Lactobacillus rhamnosus SDSP202418 on exercise-induced fatigue in mice
Source: Front Microbiol. 2024 Sep 26;15:1420872. doi: 10.3389/fmicb.2024.1420872 (PMC11464290; doi:10.3389/fmicb.2024.1420872)
Supplement: Supplementary file 1 [file Data_Sheet_1.ZIP › date/lefse_asv_table_SDSP8L_ConLvsNorLvsVcLvsSDSP18L_2.5.tar.gz!/lefse_asv_table_SDSP8L_ConLvsNorLvsVcLvsSDSP18L_2.5/ConL-vs-NorL-vs-VcL-vs-SDSP18L/biomarkers_raw_images/1_Bacteria-Bacteroidota-Bacteroidia-Bacteroidales-Bacteroidaceae-Bacteroides-Bacteroides_stercorirosoris.pdf]

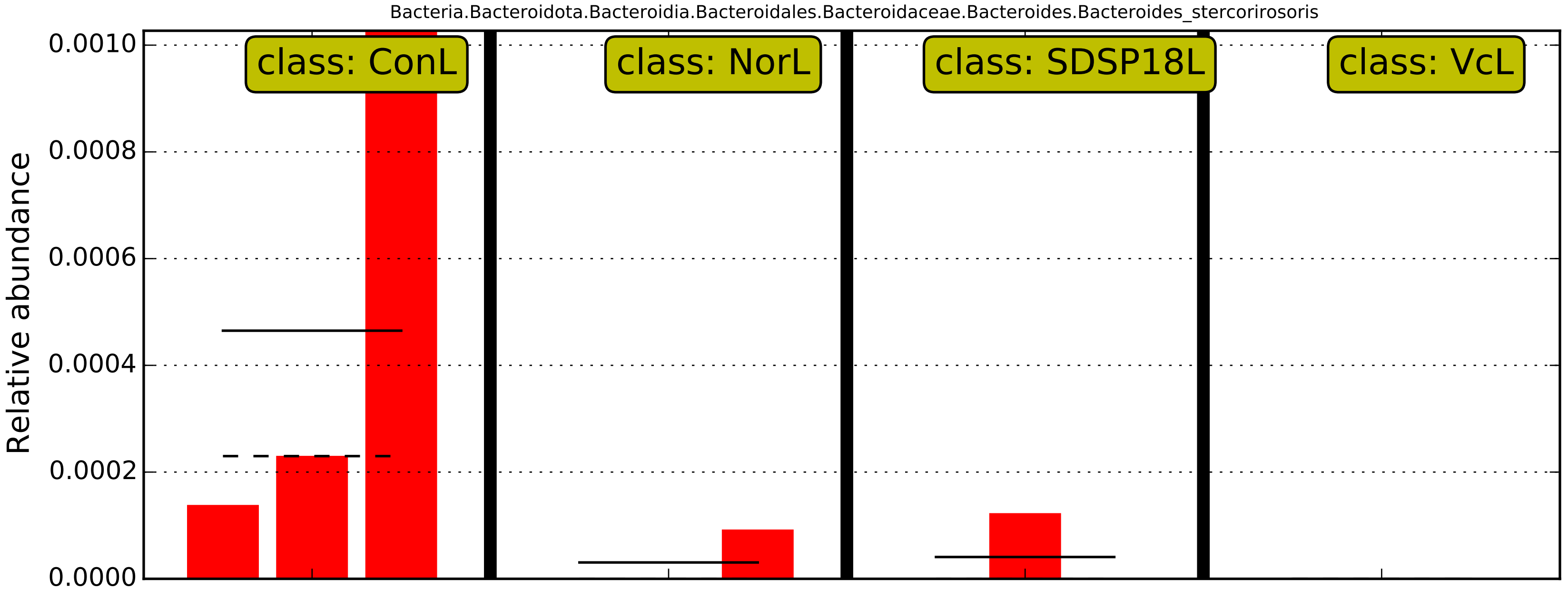

Bacteria.Bacteroidota.Bacteroidia.Bacteroidales.Bacteroidaceae.Bacteroides.Bacteroides_stercorirosoris
Relative abundance
0.0010
0.0008
0.0006
0.0004
0.0002
0.0000
class: ConL
class: NorL
class: SDSP18L
class: VcL
