# Supplementary material for: Alleviating effect of Lactobacillus rhamnosus SDSP202418 on exercise-induced fatigue in mice
Source: Front Microbiol. 2024 Sep 26;15:1420872. doi: 10.3389/fmicb.2024.1420872 (PMC11464290; doi:10.3389/fmicb.2024.1420872)
Supplement: Supplementary file 1 [file Data_Sheet_1.ZIP › date/lefse_asv_table_SDSP8L_ConLvsNorLvsVcLvsSDSP18L_2.5.tar.gz!/lefse_asv_table_SDSP8L_ConLvsNorLvsVcLvsSDSP18L_2.5/ConL-vs-NorL-vs-VcL-vs-SDSP18L/biomarkers_raw_images/1_Bacteria-Bacteroidota-Bacteroidia-Bacteroidales-Rikenellaceae-Rikenella.pdf]

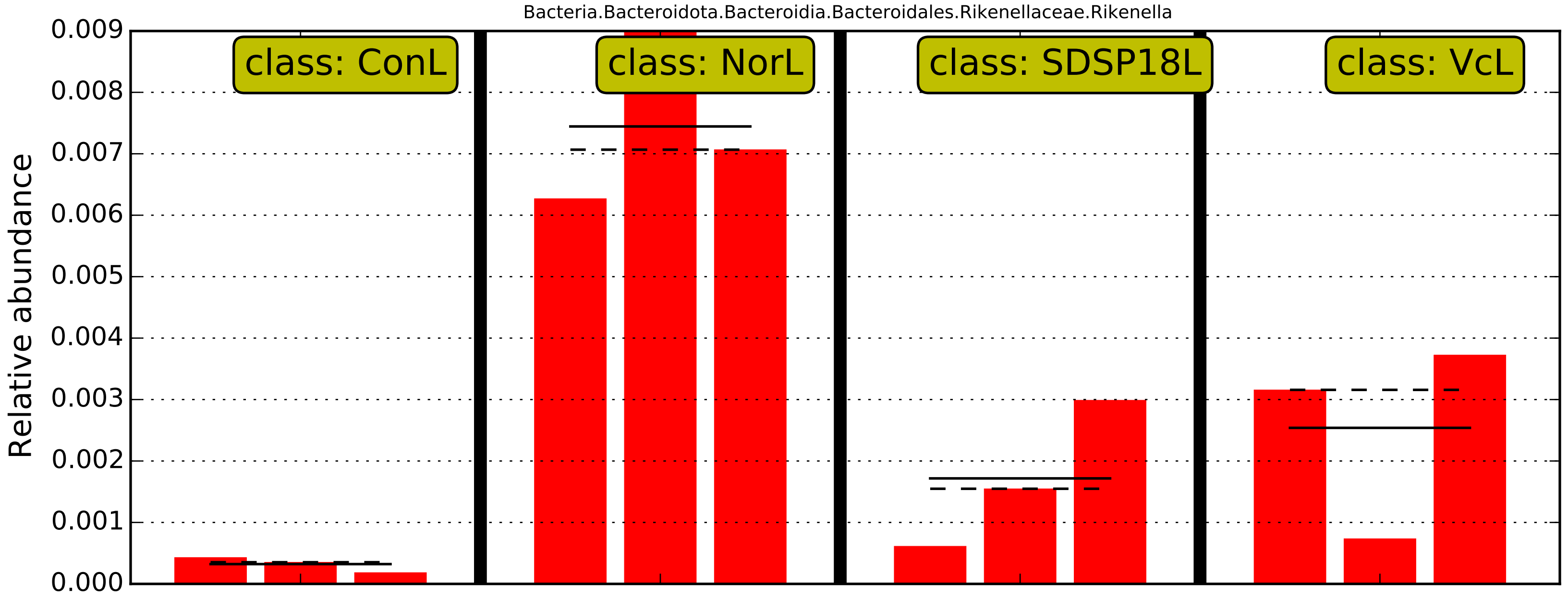

Bacteria.Bacteroidota.Bacteroidia.Bacteroidales.Rikenellaceae.Rikenella
Relative abundance
0.009
0.008
0.007
0.006
0.005
0.004
0.003
0.002
0.001
0.000
class: ConL
class: NorL
class: SDSP18L
class: VcL
