# Supplementary material for: Alleviating effect of Lactobacillus rhamnosus SDSP202418 on exercise-induced fatigue in mice
Source: Front Microbiol. 2024 Sep 26;15:1420872. doi: 10.3389/fmicb.2024.1420872 (PMC11464290; doi:10.3389/fmicb.2024.1420872)
Supplement: Supplementary file 1 [file Data_Sheet_1.ZIP › date/lefse_asv_table_SDSP8L_ConLvsNorLvsVcLvsSDSP18L_2.5.tar.gz!/lefse_asv_table_SDSP8L_ConLvsNorLvsVcLvsSDSP18L_2.5/ConL-vs-NorL-vs-VcL-vs-SDSP18L/biomarkers_raw_images/1_Bacteria-Firmicutes-Clostridia-Lachnospirales-Lachnospiraceae-Blautia.pdf]

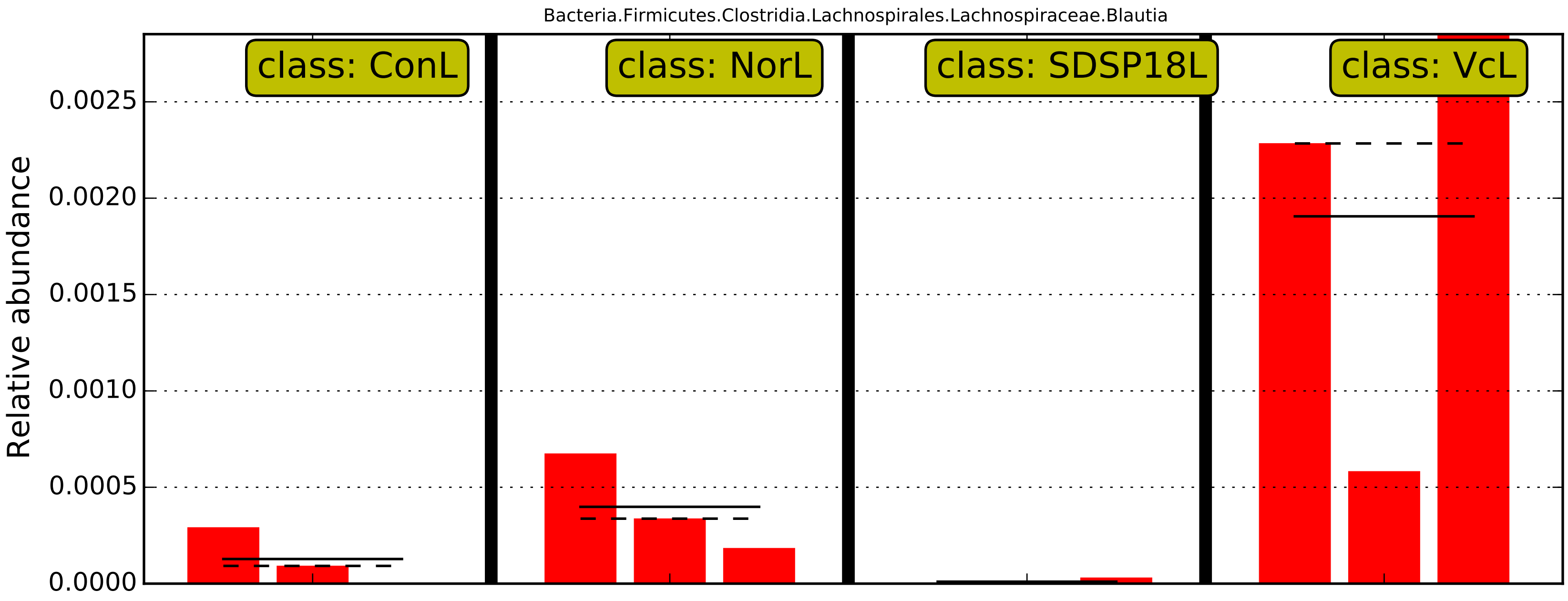

Bacteria.Firmicutes.Clostridia.Lachnospirales.Lachnospiraceae.Blautia
Relative abundance
0.0000
0.0005
0.0010
0.0015
0.0020
0.0025
class: ConL
class: NorL
class: SDSP18L
class: VcL
