# Supplementary material for: Alleviating effect of Lactobacillus rhamnosus SDSP202418 on exercise-induced fatigue in mice
Source: Front Microbiol. 2024 Sep 26;15:1420872. doi: 10.3389/fmicb.2024.1420872 (PMC11464290; doi:10.3389/fmicb.2024.1420872)
Supplement: Supplementary file 1 [file Data_Sheet_1.ZIP › date/lefse_asv_table_SDSP8L_ConLvsNorLvsVcLvsSDSP18L_2.5.tar.gz!/lefse_asv_table_SDSP8L_ConLvsNorLvsVcLvsSDSP18L_2.5/ConL-vs-NorL-vs-VcL-vs-SDSP18L/biomarkers_raw_images/1_Bacteria-Proteobacteria-Gammaproteobacteria-Burkholderiales-Nitrosomonadaceae-Nitrosomonas.pdf]

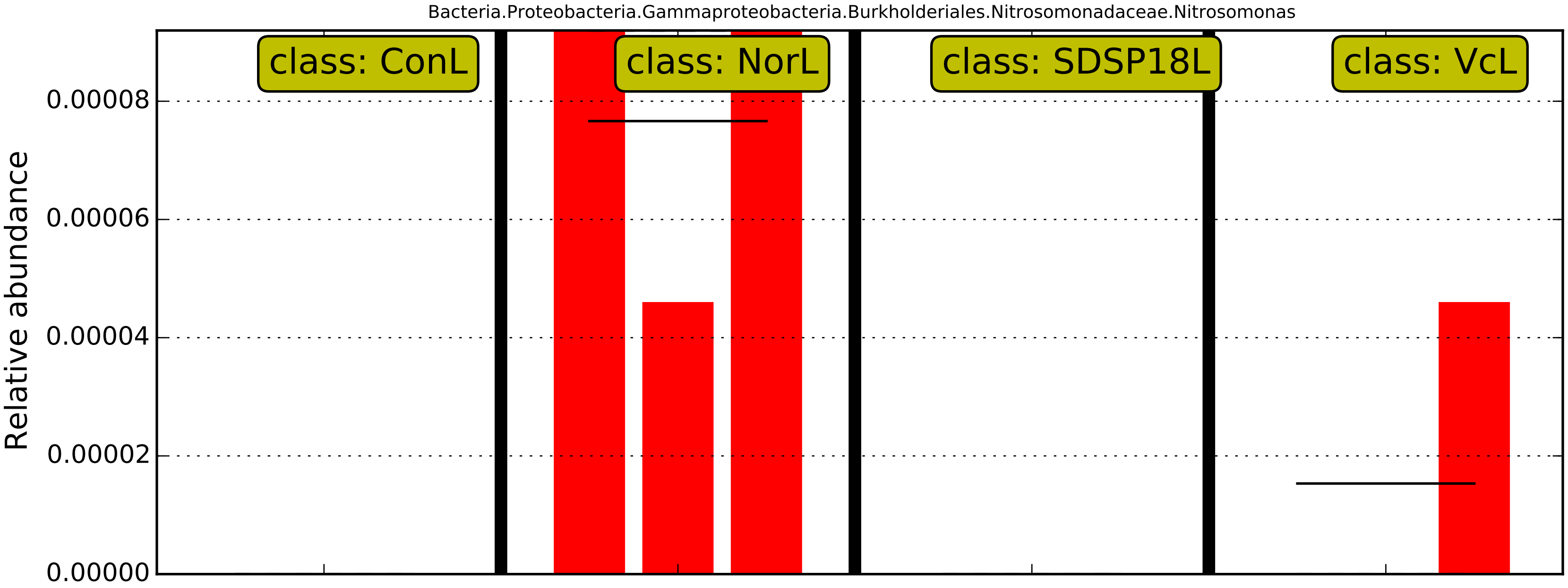

Bacteria.Proteobacteria.Gammaproteobacteria.Burkholderiales.Nitrosomonadaceae.Nitrosomonas
class: ConL
class: NorL
class: SDSP18L
class: VcL
Relative abundance
0.00008
0.00006
0.00004
0.00002
0.00000
