# Supplementary material for: Alleviating effect of Lactobacillus rhamnosus SDSP202418 on exercise-induced fatigue in mice
Source: Front Microbiol. 2024 Sep 26;15:1420872. doi: 10.3389/fmicb.2024.1420872 (PMC11464290; doi:10.3389/fmicb.2024.1420872)
Supplement: Supplementary file 1 [file Data_Sheet_1.ZIP › date/lefse_asv_table_SDSP8L_ConLvsNorLvsVcLvsSDSP18L_2.5.tar.gz!/lefse_asv_table_SDSP8L_ConLvsNorLvsVcLvsSDSP18L_2.5/single_level/class/LDA.pdf]

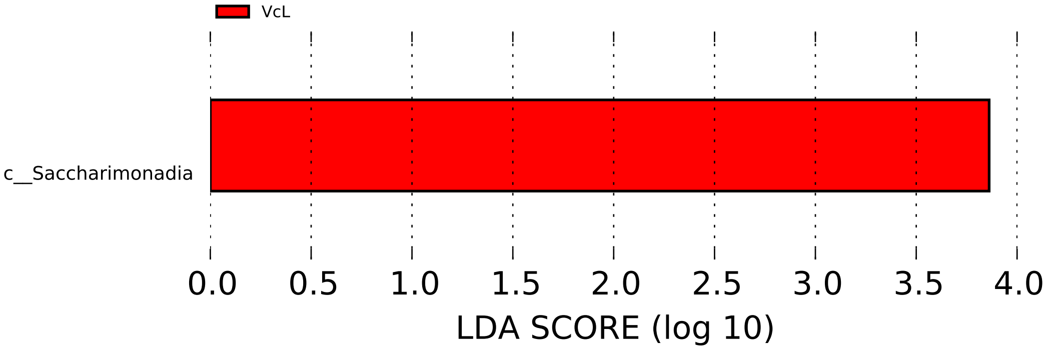
VcL
c__Saccharimonadia
0.0
0.5
1.0
1.5
2.0
2.5
3.0
3.5
4.0
LDA SCORE (log 10)
