# Supplementary material for: Alleviating effect of Lactobacillus rhamnosus SDSP202418 on exercise-induced fatigue in mice
Source: Front Microbiol. 2024 Sep 26;15:1420872. doi: 10.3389/fmicb.2024.1420872 (PMC11464290; doi:10.3389/fmicb.2024.1420872)
Supplement: Supplementary file 1 [file Data_Sheet_1.ZIP › date/lefse_asv_table_SDSP8L_ConLvsNorLvsVcLvsSDSP18L_2.5.tar.gz!/lefse_asv_table_SDSP8L_ConLvsNorLvsVcLvsSDSP18L_2.5/single_level/genus/LDA.pdf]

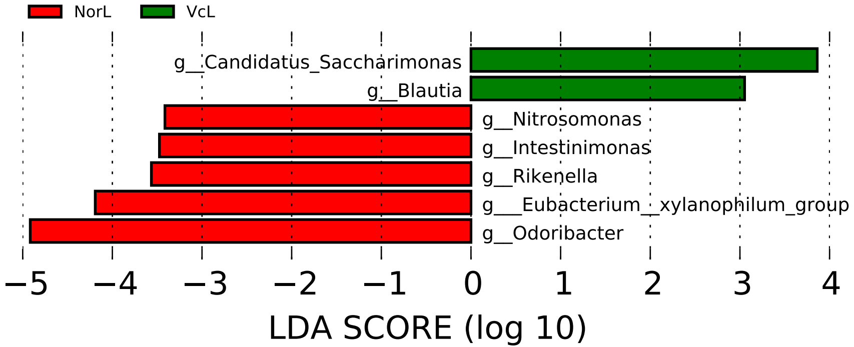
NorL
VcL
g__Candidatus_Saccharimonas
g__Blautia
g__Nitrosomonas
g__Intestinimonas
g__Rikenella
g___Eubacterium__xylanophilum_group
g__Odoribacter
−5
−4
−3
−2
−1
0
1
2
3
4
LDA SCORE (log 10)
