# Supplementary material for: Alleviating effect of Lactobacillus rhamnosus SDSP202418 on exercise-induced fatigue in mice
Source: Front Microbiol. 2024 Sep 26;15:1420872. doi: 10.3389/fmicb.2024.1420872 (PMC11464290; doi:10.3389/fmicb.2024.1420872)

Cladogram

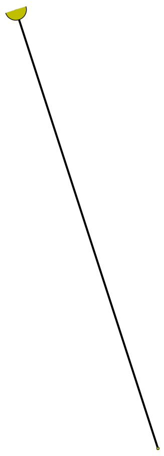

Supplement: Supplementary file 1 [file Data_Sheet_1.ZIP › date/lefse_asv_table_SDSP8L_ConLvsNorLvsVcLvsSDSP18L_2.5.tar.gz!/lefse_asv_table_SDSP8L_ConLvsNorLvsVcLvsSDSP18L_2.5/single_level/kingdom/LDA.tree.pdf]
