# Supplementary material for: Alleviating effect of Lactobacillus rhamnosus SDSP202418 on exercise-induced fatigue in mice
Source: Front Microbiol. 2024 Sep 26;15:1420872. doi: 10.3389/fmicb.2024.1420872 (PMC11464290; doi:10.3389/fmicb.2024.1420872)
Supplement: Supplementary file 1 [file Data_Sheet_1.ZIP › date/pca_asv_table_SDSP8L.tar.gz!/pca_asv_table_SDSP8L.result/pca_asv_table_SDSP8L_cluster.pdf]

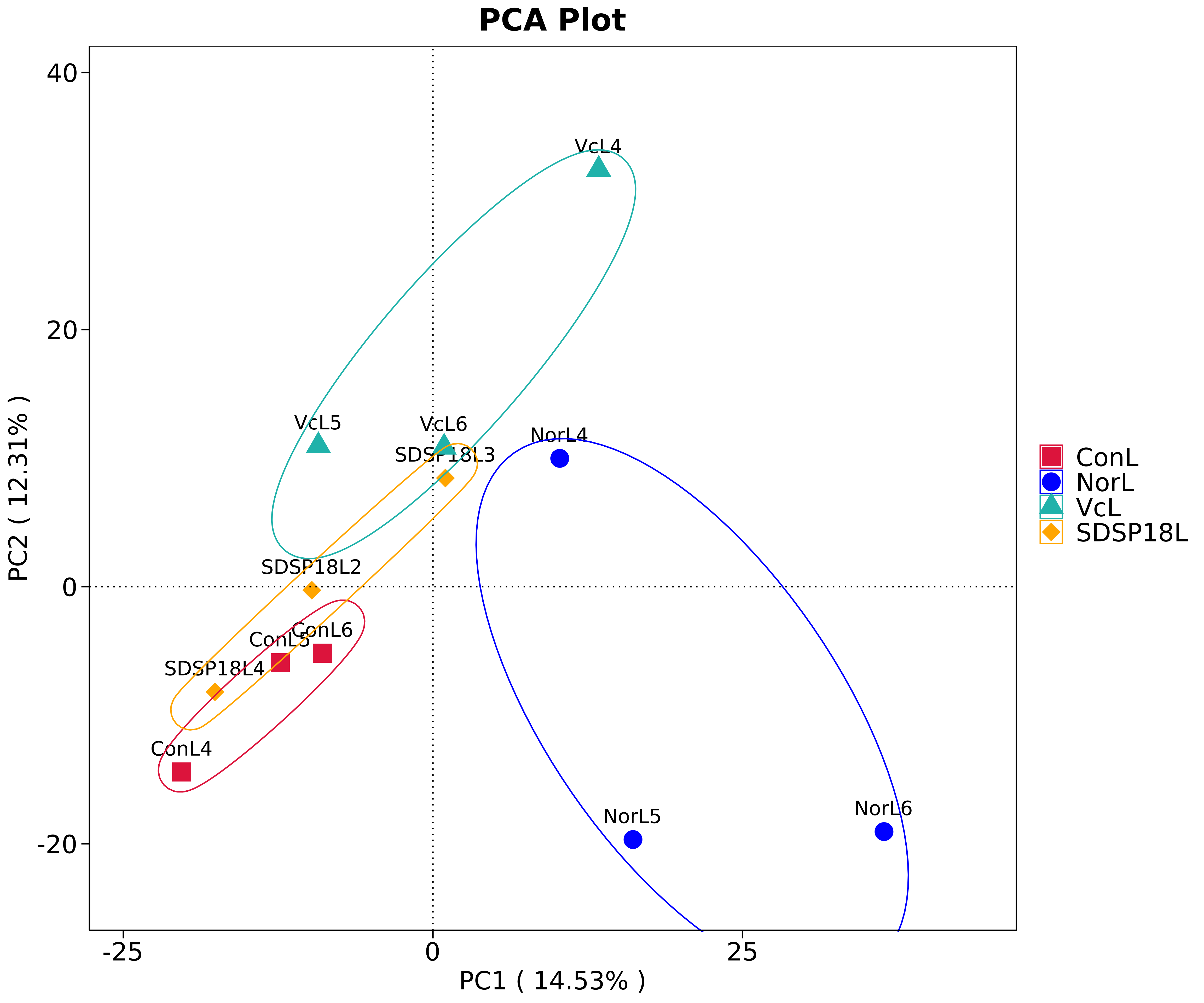

PCA Plot
VcL4
VcL5
VcL6
NorL4
SDSP18L3
SDSP18L2
ConL5
ConL6
SDSP18L4
ConL4
NorL5
NorL6
40
20
0
-20
-25
0
25
PC1 (14.53%)
PC2 (12.31%)
ConL
NorL
VcL
SDSP18L
