# Supplementary material for: Alleviating effect of Lactobacillus rhamnosus SDSP202418 on exercise-induced fatigue in mice
Source: Front Microbiol. 2024 Sep 26;15:1420872. doi: 10.3389/fmicb.2024.1420872 (PMC11464290; doi:10.3389/fmicb.2024.1420872)
Supplement: Supplementary file 1 [file Data_Sheet_1.ZIP › date/pcoa_asv_table_control.sdsp18l_unweighted_unifrac.tar.gz!/pcoa_asv_table_control.sdsp18l_unweighted_unifrac.result/pcoa_asv_table_control.sdsp18l_unweighted_unifrac_2.pdf]

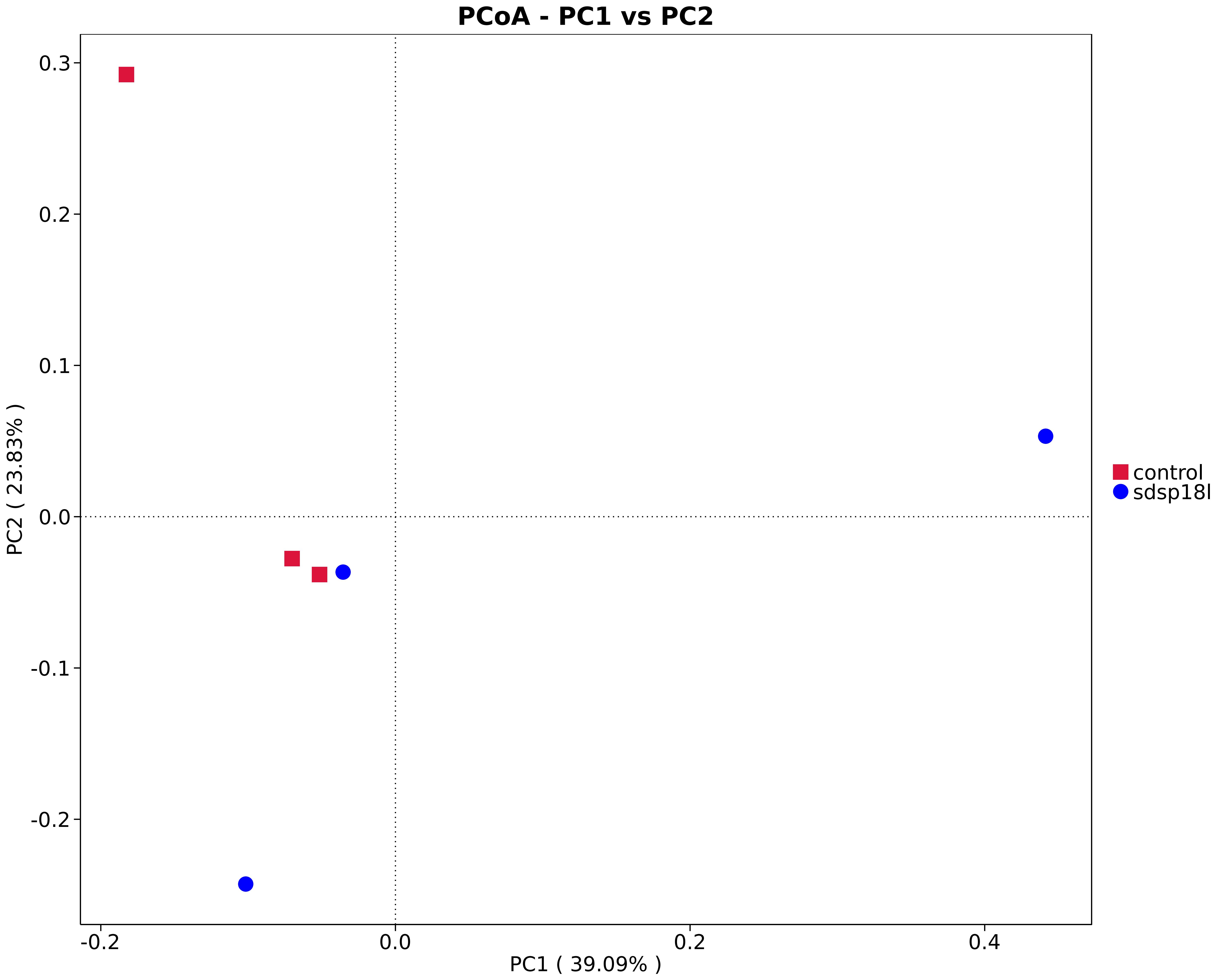
PCoA - PC1 vs PC2
0.3
0.2
0.1
0.0
-0.1
-0.2
PC2 (23.83%)
-0.2
0.0
0.2
0.4
PC1 (39.09%)
control
sdsp18l
