# Supplementary material for: Alleviating effect of Lactobacillus rhamnosus SDSP202418 on exercise-induced fatigue in mice
Source: Front Microbiol. 2024 Sep 26;15:1420872. doi: 10.3389/fmicb.2024.1420872 (PMC11464290; doi:10.3389/fmicb.2024.1420872)
Supplement: Supplementary file 1 [file Data_Sheet_1.ZIP › date/top_SDSP18L_SDSP8L_20_phylum,genus,species_group.phylum.tar.gz!/top_SDSP18L_SDSP8L_20_phylum,genus,species_group.phylum.result/top_SDSP18L_SDSP8L_20_phylum,genus,species_group.phylum_no_others.pdf]

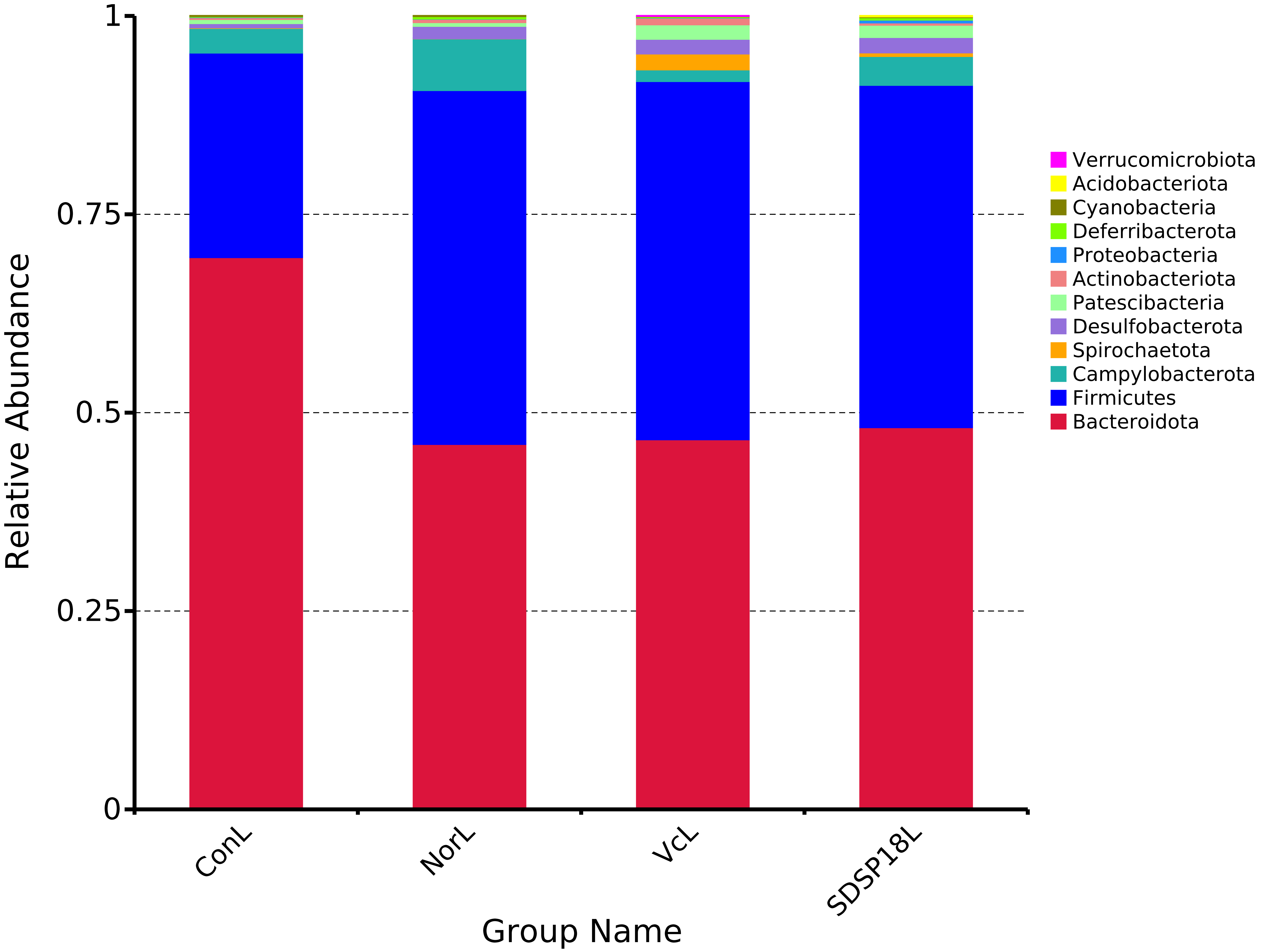
Relative Abundance
1
0.75
0.5
0.25
0
ConL
NorL
VcL
SDSP18L
Group Name
Verrucomicrobiota
Acidobacteriota
Cyanobacteria
Deferribacterota
Proteobacteria
Actinobacteriota
Patescibacteria
Desulfobacterota
Spirochaetota
Campylobacterota
Firmicutes
Bacteroidota
